# Pathological α-synuclein recruits LRRK2 expressing pro-inflammatory monocytes to the brain

**DOI:** 10.1186/s13024-021-00509-5

**Published:** 2022-01-10

**Authors:** Enquan Xu, Ravindra Boddu, Hisham A. Abdelmotilib, Arpine Sokratian, Kaela Kelly, Zhiyong Liu, Nicole Bryant, Sidhanth Chandra, Samantha M. Carlisle, Elliot J. Lefkowitz, Ashley S. Harms, Etty N. Benveniste, Talene A. Yacoubian, Laura A. Volpicelli-Daley, David G. Standaert, Andrew B. West

**Affiliations:** 1 Duke Center for Neurodegeneration Research, Duke University, Durham, NC 27710 USA; 2 Department of Pharmacology and Cancer Biology, Duke University, 3 Genome Court, Durham, NC 27710 USA; 3 Department of Neurology, University of Iowa, Iowa City, IA USA; 4 Medical Scientist Training Program, Northwestern University Feinberg School of Medicine, Chicago, IL 60611 USA; 5 Center for Clinical and Translational Science, University of Alabama at Birmingham, Birmingham, AL 35294 USA; 6 Department of Chemistry and Biochemistry, New Mexico State University, Las Cruces, NM 88003 USA; 7 Department of Microbiology, University of Alabama at Birmingham, Birmingham, AL 35294 USA; 8 Center for Neurodegeneration and Experimental Therapeutics, Department of Neurology, University of Alabama at Birmingham, Birmingham, AL 35216 USA; 9 Department of Cell, Developmental and Integrative Biology, University of Alabama at Birmingham, Birmingham, AL 35294 USA

**Keywords:** SNCA, PARK8, Monocyte extravasation, Neurodegeneration

## Abstract

**Background:**

*Leucine rich repeat kinase 2* (*LRRK2*) and *SNCA* are genetically linked to late-onset Parkinson’s disease (PD). Aggregated α-synuclein pathologically defines PD. Recent studies identified elevated LRRK2 expression in pro-inflammatory CD16+ monocytes in idiopathic PD, as well as increased phosphorylation of the LRRK2 kinase substrate Rab10 in monocytes in some *LRRK2* mutation carriers. Brain-engrafting pro-inflammatory monocytes have been implicated in dopaminergic neurodegeneration in PD models. Here we examine how α-synuclein and LRRK2 interact in monocytes and subsequent neuroinflammatory responses.

**Methods:**

Human and mouse monocytes were differentiated to distinct transcriptional states resembling macrophages, dendritic cells, or microglia, and exposed to well-characterized human or mouse α-synuclein fibrils. LRRK2 expression and LRRK2-dependent Rab10 phosphorylation were measured with monoclonal antibodies, and myeloid cell responses to α-synuclein fibrils in R1441C-Lrrk2 knock-in mice or G2019S-Lrrk2 BAC mice were evaluated by flow cytometry. Chemotaxis assays were performed with monocyte-derived macrophages stimulated with α-synuclein fibrils and microglia in Boyden chambers.

**Results:**

α-synuclein fibrils robustly stimulate LRRK2 and Rab10 phosphorylation in human and mouse macrophages and dendritic-like cells. In these cells, α-synuclein fibrils stimulate LRRK2 through JAK-STAT activation and intrinsic LRRK2 kinase activity in a feed-forward pathway that upregulates phosphorylated Rab10. In contrast, LRRK2 expression and Rab10 phosphorylation are both suppressed in microglia-like cells that are otherwise highly responsive to α-synuclein fibrils. Corroborating these results, LRRK2 expression in the brain parenchyma occurs in pro-inflammatory monocytes infiltrating from the periphery, distinct from brain-resident microglia. Mice expressing pathogenic *LRRK2* mutations G2019S or R1441C have increased numbers of infiltrating pro-inflammatory monocytes in acute response to α-synuclein fibrils. In primary cultured macrophages, LRRK2 kinase inhibition dampens α-synuclein fibril and microglia-stimulated chemotaxis.

**Conclusions:**

Pathologic α-synuclein activates LRRK2 expression and kinase activity in monocytes and induces their recruitment to the brain. These results predict that LRRK2 kinase inhibition may attenuate damaging pro-inflammatory monocyte responses in the brain.

**Supplementary Information:**

The online version contains supplementary material available at 10.1186/s13024-021-00509-5.

## Background

Rare missense pathogenic mutations in the *leucine-rich repeat kinase 2* (*LRRK2*) gene that cause Parkinson’s disease (PD) upregulate LRRK2 kinase activity [1, 2]. *LRRK2* is independently associated with PD through genome-wide association studies [3, 4], as well as with Crohn’s disease and mycobacterium infection susceptibility [5]. Common genetic variation in the *LRRK2* locus that associates with PD risk links to the quantitative expression of pro-inflammatory genes in monocytes [6]. In humans, *LRRK2* is principally expressed in blood (gtexportal.org), and LRRK2 protein levels are elevated in classical (e.g., pro-inflammatory) monocyte populations as measured in idiopathic PD patients (lacking pathogenic *LRRK2* mutations) in two different study populations in Europe and the United States [7, 8]. The cause of upregulated LRRK2 levels in monocytes in PD is not known, and *LRRK2* transcriptional regulation and control of downstream LRRK2 kinase activity is not well understood in monocytes.

In monocyte-derived macrophages, LRRK2 expression and activity upregulates chemotaxis and AKT-dependent chemokine receptor signaling through phosphorylation of the LRRK2-kinase substrate Rab10 [9–11]. PD-associated mutations in *LRRK2*, as well as mutated VPS35, upregulate LRRK2-dependent Rab10 phosphorylation in monocytes [12]. Considering the potential role for LRRK2 in chemotaxis, brain-engrafting monocyte-derived macrophages have been previously implicated in the destruction of dopaminergic neurons in the rAAV2-α-synuclein expression model and the toll-like receptor (TLR)-4 agonist lipopolysaccharide (LPS) model [13, 14]. In the rat brain, Lrrk2 protein colocalizes inside activated CD68+ myeloid cells of unknown origins, recruited in response to rAAV2-α-synuclein expression or intracranial injections of LPS [15]. In the pre-formed α-synuclein fibril model, immune cell recruitment to the brain may occur early after fibril injection, persists for months, and causes a notable systemic upregulation of pro-inflammatory cells [16, 17]. In both the AAV and fibril models, owing to the broad overlap and dynamic nature of protein and lipid markers used to identify different types of myeloid cells, the identity of the specific innate immune cell compartments where LRRK2 might exert intrinsic function to potentially modify neuroinflammatory phenotypes in disease has not been clear.

The principle sources of innate immune cells in both human and rodent brains include abundant resident microglia as well as non-resident macrophages found in the perivasculature and meningeal spaces [18]. Based on past studies that demonstrate robust LRRK2 effects on microglial responses both in cell culture and in mouse models [19–22], it is widely presumed that intrinsic LRRK2 expression in microglia drives immunological phenotypes in the brain. Potentially at odds with this assumption, *Lrrk2* expression is relatively low or sometimes undetectable in the sizable population of sentinel microglia in rodent or human brain as assessed by recent single-cell sequencing efforts [23, 24] or through immunohistochemistry [25, 26]. However, other studies have convincingly demonstrated LRRK2 function in isolated microglia cells in culture, potentially functioning through NFATc2 and toll-receptor signaling pathways [19, 21, 27, 28].

Here, we seek to resolve the different types of immune cells harboring LRRK2 protein expression and Rab10 phosphorylation in response to PD-associated aggregated α-synuclein. Using a powerful comparative strategy developed by Ryan et al. to polarize human blood-derived monocytes to distinct transcriptional states [29], in complement with observations in vivo in mouse models, our results show that LRRK2 protein levels, kinase activity, and induction by pathological α-synuclein are largely restricted to monocyte-derived macrophages, distinct from microglia. In mice, LRRK2-kinase activating mutations G2019S and R1441C tend to enhance the recruitment of pro-inflammatory monocyte-derived macrophages to the brain, in immune cells that express the highest levels of LRRK2 protein, consistent with a role for LRRK2 in chemotaxis observed in cell culture models. These results provide evidence that LRRK2 may drive neuroinflammatory responses in disease in part through promoting the recruitment of pro-inflammatory monocytes to the brain.

## Methods

### Human cell cultures

Blood mononuclear cells were isolated from venous blood draws from healthy volunteers. Healthy volunteers in this study (age range 24–34) did not have a family history of PD, did not report ancestries known to be enriched with pathogenic *LRRK2* mutations, and were not taking anti-inflammatory medications. Blood draws were processed with SepMate (Fisher) tubes and Lymphoprep (Stemcell Tech) tubes according to manufacturer’s recommendations and previous reports [29]. EasySep Negative Selection Human Monocyte Enrichment kits (Stemcell Tech), without CD16 depletion, were used according to manufacturer’s recommendations. Macrophage cells (i.e., monocyte-derived macrophages, MDMs) were cultured in DMEM (Invitrogen) supplemented with Glutamax (Invitrogen), 10% fetal bovine serum (FBS), 1% penicillin/streptomycin (Invitrogen), Fungizone (2.5 g・mL^− 1^), and human MCSF (20 ng・mL^− 1^). For dendritic-like cells and microglia-like cells [29], media included RPMI-1640 (Invitrogen), Glutamax (Life-Technologies), 1% penicillin/streptomycin (Invitrogen), and Fungizone (2.5 g・mL^− 1^; Life Technologies). Dendritic-like cells were further supplemented with 10% FBS (Bio-Techne) and human GMCSF (5 ng・mL^− 1^). Microglia-like cells were supplemented with MCSF (10 ng・mL-1), GMCSF (10 ng・mL^− 1^), NGF-β (10 ng・mL^− 1^), CCL2 (100 ng・mL^− 1^), and IL-34 (100 ng・mL^− 1^). All cytokines were purchased from PeproTech. All cells were cultured for at least 1 week before experiments. As indicated, some cells were activated with LPS (100 ng・mL^− 1^ triple-purified from *E. coli* strain O55:B5, InvivoGen) for 48 h. Alternatively-activated cells, as indicated, were generated with the addition of 20 ng・mL^− 1^ IL-4 and IL-13 for 48 h.

### Mice and mouse cell culture

Lrrk2 R1441C-KI (JAX Stock # 009346), Lrrk2 WT-BAC transgenic mice (JAX stock #012466), Lrrk2 G2019S-BAC (JAX stock #012467), Lrrk2 knock-out (KO) and C57BL/6 J WT (nTg) control mice (JAX stock #000664) were obtained from Jackson Laboratories. Mice were genotyped according to published protocols by the depositing investigators, including quantitative PCR protocols used for measuring BAC copy numbers through the course of the project. Mouse bone marrow-derived cells were procured from 3 to 5 months-old mice. Bone marrow cells were flushed with ice-cold PBS and transferred through 70 μm nylon cell strainers (Millipore). Cells were centrifuged at 450 xg for 10 min at 4 °C and incubated with red blood cell lysis buffer (Invitrogen) for 30 s. Cells were dissociated and cultured with DMEM media supplemented with 10% FBS and 20 ng・mL^− 1^ mouse MCSF (PeproTech). The immortalized mouse microglial cell line BV-2 (ATCC CRL-2469) was cultured as previously described [30].

For chemotaxis experiments, primary mouse bone marrow-derived macrophages were applied to the upper chamber of Transwell dishes (Corning Costar, CLS3464), and fibril-conditioned media was added into the lower chamber at a 1 to 10 (w/v) ratio. Two hours post-conditioned media addition, non-mobile cells in the upper chamber were removed, washed, and the upper surface layer swabbed with cotton. The migrated cells at the bottom surface of the Transwell membrane were fixed with 4% paraformaldehyde and stained with DAPI for automated counting on a Keyence BZ-X800 microscope.

### Recombinant α-synuclein

Bacterial expression plasmids encoding mouse and human WT-α-synuclein in the inducible pRK172 backbone were transformed into BL21-CodonPlus (DE3) cells (Clontech). Cell pellets were lysed in 0.75 M NaCl, 10 mM Tris-HCl, pH 7.6, 1 mM EDTA, 1 mM PMSF and sonicated at 70% power (Fisher500 Dismembrator) for 1 min, and tubes were placed in boiling water for 15 min. Centrifuged samples were dialyzed against 10 mM Tris (pH 7.6) with 50 mM NaCl, 1 mM EDTA, 1 mM PMSF. The suspension was passed through a HiPrep Q HP 16/10 column (GE Healthcare) on an ÄKTA pure protein purification system (Cytiva) with a running buffer composed of 10 mM Tris pH 7.6, 25 mM NaCl, and eluted with a linear gradient application of high-salt buffer (10 mM Tris, 1 M NaCl pH 7.6). Samples containing single-band profiles of α-synuclein were identified by Coomassie staining and further dialyzed and concentrated. Monomer protein passed through three rounds of endotoxin removal (Endotoxin removal kit, GenScript) to reach a low level of < 0.1 E.U.・mg^− 1^, with endotoxin levels determined using a LAL chromogenic endotoxin quantification kit (GenScript). Mouse or human α-synuclein fibrils were prepared through incubation of 7 mg・mL^− 1^ α-synuclein monomer in phosphate-buffer saline for 7 days at 37 °C with constant agitation. Fibrils were washed and subjected to a second round of amplification as described [31]. Final sonicated preparations were measured by dynamic light scattering on a Titan DynaPro (Wyatt Technology), with molecular weights of terminal particles estimated from A_280_, intensity, and mass distributions, as described [31].

### Intracranial stereotaxic injections

Consistent with past studies [32], 5 μg・mL^− 1^ preparations of human α-synuclein fibrils or monomer (2 μL per site) were injected into ∼8–16 weeks old mice. 30-gauge needles (Hamilton) with a 110° bevel were used for injections with solutions infused using an automated stereotaxic injector (Stoelting) at a flow rate of 0.25 μL per min with the bevel of the needle facing medially according to stereotaxic coordinates with respect to Bregma (AP − 3.1 mm, ML ± 1.5, DV − 4.6 mm). At the indicated time of sacrifice, mice were transcardially perfused with cold PBS (pH 7.4) and brains removed and dissected for flow cytometry.

### Flow cytometry

Whole brains were procured from mice perfused with cold PBS and digested with 1 mg・mL^− 1^ collagenase IV (Sigma) with 20 μg・mL^− 1^ DNAse I (Sigma) diluted in RPMI-1640 with 10% FBS, 1% L-glutamine, and 1% penicillin-streptomycin (Invitrogen) as described [13, 33]. Mononuclear cells were isolated with a 30/70% Percoll gradient and blocked with Fcγ receptor antibody clone 2.4G2. Leukocytes from the spleen were isolated as previously described [34]. Anesthetized mice were perfused with cold PBS and minced spleens were digested with Liberase DL (Roche Diagnostics, Inc.) in DMEM (30 min at 37 °C with shaking). Single-cell suspension from splenic tissues were prepared by passing through 18 Ga. and 20 Ga. needles followed by passage through a 40-μm nylon filter. An Ammonium-Chloride-Potassium (ACK) lysis buffer (Invitrogen) was used for lysing red blood cells. For some experiments, mouse midbrains were dissected from whole brain dissections from a 2 mm-thick coronal slice, with the midbrain carefully removed, minced, and tissue pieces triturated and digested in 1 mg・mL^− 1^ collagenase IV with 20 μg・mL^− 1^ DNAse I diluted in HBSS (Invitrogen) for 35 min at 37 °C in a water bath with gentle intermittent shaking. The digestion was halted with incubation in PBS, 2 mM EDTA, and 1% w/v bovine-serum albumin (Sigma) on ice.

Antibody clones were obtained from eBioscience, BioLegend, BD Pharm, Abcam and Life Technologies and included CD45 brilliant violet (BV) 650 (clone 104); CD45 BV650 (clone 30-F11); CD45 eFluor450 (clone 30-F11); CD11b eVolve 605 (clone M1/70); Ly6G allophycocyanin-conjugated (APC) (Gr-1, clone 1A8); Ly6C eFluor(eF)450 (clone HK1.4); CD11b BV605 (clone M1/70); CD11b phycoerythrin (PE) (clone M1/70); MHCII FITC (I-A/I-E); CD4 PE-Cyanine7 (clone GK1.5); CD8a APC-Cyanine7 (clone 53–6.7) and rabbit monoclonal [MJFF2 (c41–2)] anti-LRRK2 (AlexaFluor 647), in addition to 7-aminoactinomycin D (7-AAD) for live-dead staining and fixable viability dye (Live/Dead Fixable Dead Cell Stain Kit, Near IR). All cell suspensions were analyzed on an Attune NxT instrument (acoustic-assisted hydrodynamic focusing cytometer; Thermo Fisher Scientific) and all results/cytometer acquired data were analyzed using FlowJo 10.7 software (BD Company).

Flow cytometry for detecting intracellular LRRK2 in mouse cells was accomplished according to Bliederhaeuser and Cook with several modifications [7, 8]. The two-step protocol for intracellular (cytoplasmic) proteins was performed with the Intracellular Fixation and Permeabilization Buffer set (eBioscience) according to manufacturer’s recommendations with the rabbit monoclonal [MJFF2 (c41–2)] anti-LRRK2 antibody conjugated to Alexa Fluor 647. Alexa Fluor647 median fluorescence intensity (MFI) signals from Lrrk2 KO mice were subtracted from each cell population processed in parallel. All experiments were analyzed with FlowJo 10.7 software (BD Company).

### Immunoblotting and immunofluorescence

Tissues were collected following transcardial perfusion with cold PBS, and tissues (or cells in culture) were homogenized with probe-tip sonication in RIPA lysis buffer containing 50 mM Tris (pH 7.4), 150 mM NaCl, 1% Triton, and 0.1% SDS supplemented with 1x Complete protease and PhosStop inhibitor tablets (Roche). Homogenates were centrifuged and soluble supernatants analyzed using the BCA assay (Pierce). Lysates were analyzed on 4–20% gradient mini-PROTEAN TGX stain-free gels (BioRad) and transferred to Immobilon-FL PVDF membrane (Millipore), followed by immunoblotting with the indicated primary and secondary antibodies. The following antibodies were used: N241/34 anti-LRRK2 (Antibodies Inc), phospho-T73-Rab10 (MJF-R21, Abcam), total Rab10 antibody (MJF-R23, Abcam), pY701-Stat1 (Tyr701, D4A7, CST), total Stat1 (42H3, CST), loading control Hsc70 (Cell Signaling), donkey anti-mouse 680LT (LiCor), and goat anti-rabbit HRP (Jackson Immuno). For relative quantifications, the mean of the selected experimental control group is defined as the reference value after normalization to β-actin levels in the same lane. All signals were captured digitally using a Chemidoc MP Imaging System (BioRad). Quantifications were performed using Image Lab 6.0.1 software (BioRad).

Confocal images of immunostained cells, prepared as previously described [9], were captured either on a Zeiss 880 AiryScan confocal microscope, and brightfield images were captured on an Olympus BX61 microscope. Confocal images were acquired using Zen software (Zeiss), with contrast and color balance adjusted equally across all images in the group in Adobe Illustrator. Original images are available upon request from the Authors.

### Small molecule inhibitors

All compounds were > 98.5% pure as assessed by LC/MS and NMR analysis. The small molecule LRRK2 inhibitor MLi2 was synthesized for this study using the previously described strategy by Pharmaron, Inc. [35]. AZD1480 is an ATP-competitive JAK1/2 inhibitor with an IC_50_ of 0.26 nM (Selleckchem, Inc). Tofacitinib citrate (CP-690550, Selleckchem, Inc) is an inhibitor for JAKs with an IC_50_ of 1 nM, 20 nM and 112 nM IC_50_ against JAK3, JAK2, and JAK1, respectively. Ruxolitinib (Selleckchem, Inc) is a selective JAK1/2 inhibitor with an IC_50_ of 3.3 nM/2.8 nM, respectively.

### Transcriptomic analysis

Nucleic acids were collected with TRIzol (Invitrogen) reagent. Total RNA was extracted with the Qiagen RNeasy Plus Universal mini kit following manufacturer’s instructions (Qiagen, Germany), and extracted RNA samples were quantified using Qubit 2.0 Fluorometer (Life Technologies, USA) and RNA integrity > 0.9 was verified with an Agilent TapeStation 4200 (Agilent Technologies, USA). Libraries were prepared with the NEBNext Ultra RNA Library Prep Kit according to manufacturer’s instructions (NEB, Inc.). Sequencing libraries were pooled and clustered on a single lane of a flow cell on an Illumina HiSeq instrument according to manufacturer’s instructions. Samples were sequenced using a 2 × 150 bp paired end configuration, with image analysis and base calling conducted with HiSeq Control Software (Illumina). Raw sequence data files were converted to fastq files and de-multiplexed using bcl2fastq 2.17 software. One mismatch was allowed for index sequence identification. Quality of the sequencing reads were interrogated with FastQC (version 0.11.5), and aligned with STAR aligner (version 2.5.2) using basic 2-pass mapping, with all 1st pass junctions inserted into the genome indices to GENCODE release 31 GRCh38.p12 genome using the GENCODE v31 transcript annotation, with an average of 94% of reads aligning, giving an average of 46.8 million uniquely aligned reads per sample presented in this study. Analysis and visualization of the resulting data were performed using R version 4.0.0 software. Aligned reads were quantified using the GenomicAlignments package (Bioconductor) with the “IntersectionStrict” setting. Principal component analysis (PCA) in the R environment was conducted by singular value decomposition of the centered data matrix on the most 1000 variable genes in the entire dataset. The loadings of the first (x-axis) and second (y-axis) principal components were plotted.

### Other statistics and rigor

Experiments described were independently repeated at least three times and bar graphs show mean values with SEM error bars. Data points for in vivo experiments represent individual mice. All raw data are available as Supplemental Information. Uncropped western blot images collected from the ChemiDoc analysis are available as Supplemental Information. Group statistical analyses were conducted using Graphpad Prism 8.0 software. Continuous data with more than two independent groups were evaluated for significance using a one- or two-factor ANOVA, with the indicated post-hoc test. Two-tailed, unpaired *t*-tests were used to determine the significance between two groups. All tests were performed using a significance level of α = 0.05 with 95% confidence. All groups are presented with mean values and error bars representing SEM. Samples were processed without respect to group assignment, and final data were curated before sample assignment to groups. Mice were randomized with respect to genotype and sex across all experiments.

## Results

### ɑ-synuclein fibrils induce LRRK2 expression and Rab10 phosphorylation in mouse and human monocyte-derived macrophages

**Fig. 1:**
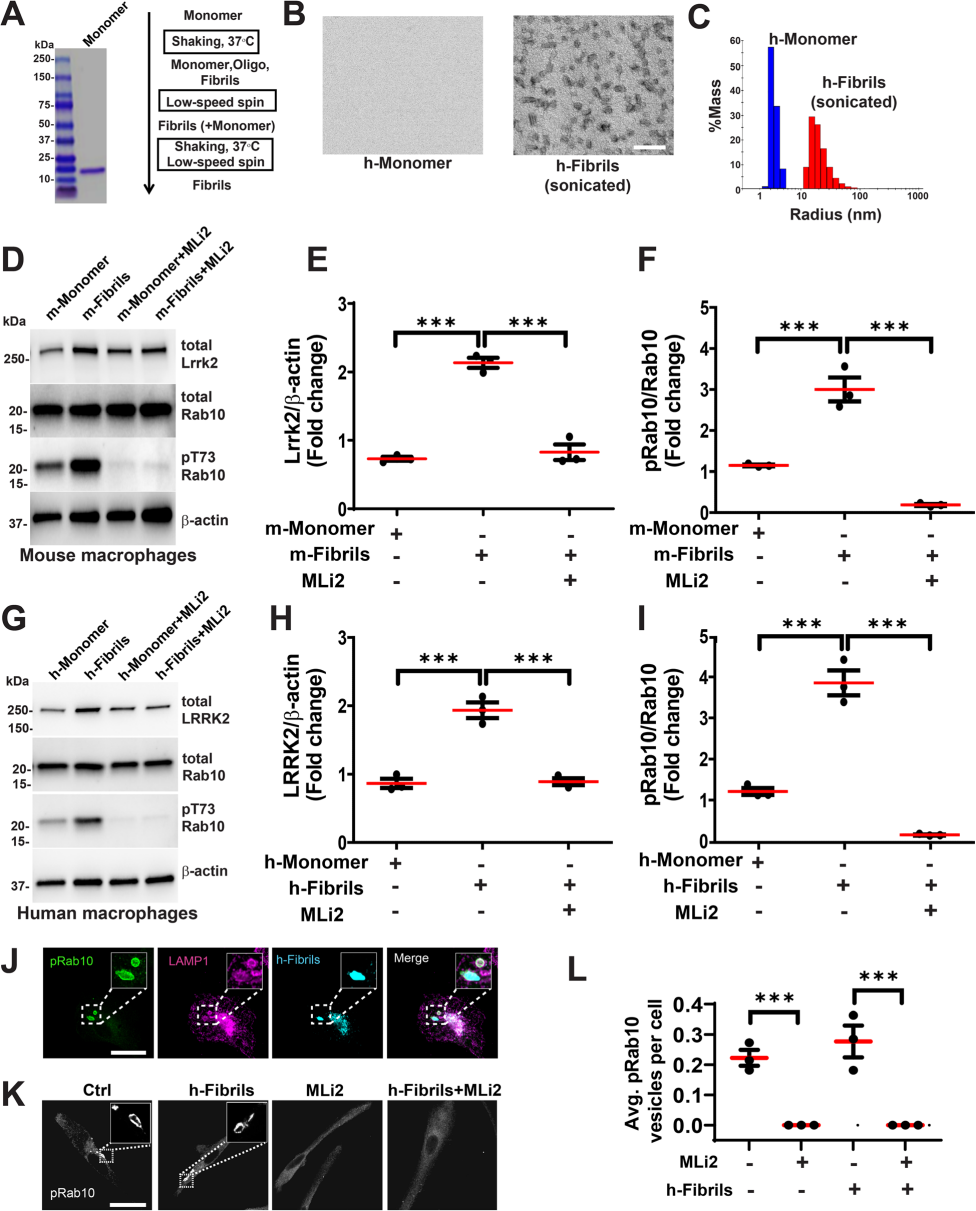
α-Synuclein fibrils induce Lrrk2/LRRK2 in monocyte-derived macrophages. **A** α-Synuclein purification and short-rod fibril construction. Preparations were verified to have low or no detectable endotoxins (see Methods). Additional quality control data are presented in Supplemental Fig. 1. **B** Electron-microscopy photomicrographs of representative human α-synuclein monomers and sonicated α-synuclein fibrils. Scale bar is 100 nm. **C** Representative dynamic light-scattering characterization of matched monomer and fibril preparations. **D** Representative immunoblots of mouse (male nTg C57Bl/6 J) bone-marrow derived macrophages, expanded with MCSF, exposed to mouse fibrils (~ 0.7 nM, 1 μg・mL^− 1^, see Methods), or mouse monomer (~ 70 nM, 1 μg・mL^− 1^), with or without the LRRK2 inhibitor MLi2 (100 nM). Results from the quantification of three independent experiments for **E** Lrrk2 protein and **F** pT73-Rab10 levels (ratio of pT73-Rab10 to total Rab10 protein). **G** Representative immunoblots of human (two healthy males and one healthy female, age range 24 to 34 years old) monocyte-derived macrophages (MCSF expanded) with quantification from three independent experiments for **H** LRRK2 protein and **I** pT73-Rab10 levels. **J** Representative confocal images of pT73-Rab10, LAMP1, and pHRodo-labeled human α-synuclein fibrils (1 μg・ mL^− 1^) in human macrophages, **K** with or without MLi2 (100 nM). Scale bars are 50 μm. **L** Quantification of the average number of pT73-Rab10 vesicles per cell. Each dot represents the mean vesicles per cell of images procured from three independent experiments, with ~ 75% of cells in control wells not showing any pT73-Rab10 vesicles, consistent with past observations in macrophages [9]. All data are group means ± SEM from *n* = 3 biologically independent experiments. Significance is determined by one-way ANOVA with Tukey’s post hoc test and ***p < 0.001

Recent studies suggest neuronal α-synuclein inclusions are composed in part of α-synuclein fibrils intermixed with membranous organelles [36, 37]. Recombinant α-synuclein fibrils, but not recombinant monomeric or oligomeric protein, potently stimulate innate immune cells [13, 17]. To determine whether α-synuclein fibrils might affect LRRK2 expression and kinase activity in immune cells, assessed inpart through measures of the LRRK2 kinase substrate Rab10 [9], cultured mouse bone-marrow derived macrophages were combined with low concentrations of mouse α-synuclein fibrils (~ 0.7 nM), or the equivalent amount of monomeric α-synuclein protein (~ 70 nM) as a control (Fig. 1A-C and Supplemental Fig. 1). In these preparations of recombinant α-synuclein, contaminating endotoxin was measured as less than 0.001 endotoxin units (E.U.) per mL. Previously, we used this concentration of fibrils as one of the lowest concentrations required to seed new inclusions in neurons in culture [38, 39]. Fibrils, but not monomeric protein, induced Lrrk2 protein expression (~ 2.5 fold, Fig. 1D, E) as well as increased pT73-Rab10 levels (~ 3 fold, Fig. 1F). Treatment with the LRRK2 kinase inhibitor, MLi2 (100 nM), interrupted α-synuclein fibril-induction of total Lrrk2 protein expression and reduced pT73-Rab10 levels (Fig. 1D-F). The ATP-competitive MLi2 molecule is selective and not known to interact with other kinases or proteins at this concentration [35, 40, 41]. In macrophages without α-synuclein fibril stimulation, MLi2 exposure did not reduce basal levels of total Lrrk2 protein, or Lrrk2 levels after incubation with α-synuclein monomers (Fig. 1D-F). Thus, the MLi2 inhibitor itself does not reduce Lrrk2 levels in macrophages, but prevents α-synuclein-fibril induced stimulation of Lrrk2.

In primary human monocyte-derived macrophages cultured from human healthy controls, the application of human α-synuclein fibrils, prepared in a similar manner to the mouse α-synuclein fibrils [31], led to a similar induction of LRRK2 protein expression as observed in mouse macrophages (~ 2 fold, Fig. 1G, H), as well as increased pT73-Rab10 levels (~ 3.8 fold, Fig. 1I). Consistently, LRRK2 kinase inhibition, through MLi2 exposure, appeared equally effective against both mouse and human LRRK2, blocking α-synuclein-induced upregulation of LRRK2 protein expression and pT73-Rab10 levels (Fig. 1G-I). Previously, we identified that pT73-Rab10 protein in macrophages specifically localized to signaling endosomes, often co-positive for CCR5-receptors and LAMP1 [9]. In the context of α-synuclein fibril exposure, pT73-Rab10 vesicles were similarly LAMP1 positive and appeared co-positive with exogenously-applied labeled (pH-Rhodo) α-synuclein fibrils (Fig. 1J). MLi2 completely ablated the detection of pT73-Rab10 vesicles visualized by immunofluorescence (Fig. 1K). While levels of pT73-Rab10 increased in lysates after fibril administration as measured by immunoblotting, α-synuclein fibril administration did not have a noticeable effect on increasing the total number of pT73-Rab10 vesicles per cell (Fig. 1L). These results suggest the amount of Rab10 phosphorylated on vesicles increases with fibrils of either mouse or human origin, is dependent on LRRK2 kinase activity, and occurs without an increase in the overt number of vesicles in the macrophage cytoplasm.

### ɑ-synuclein fibrils activate LRRK2 through JAK-STAT signaling

**Fig. 2:**
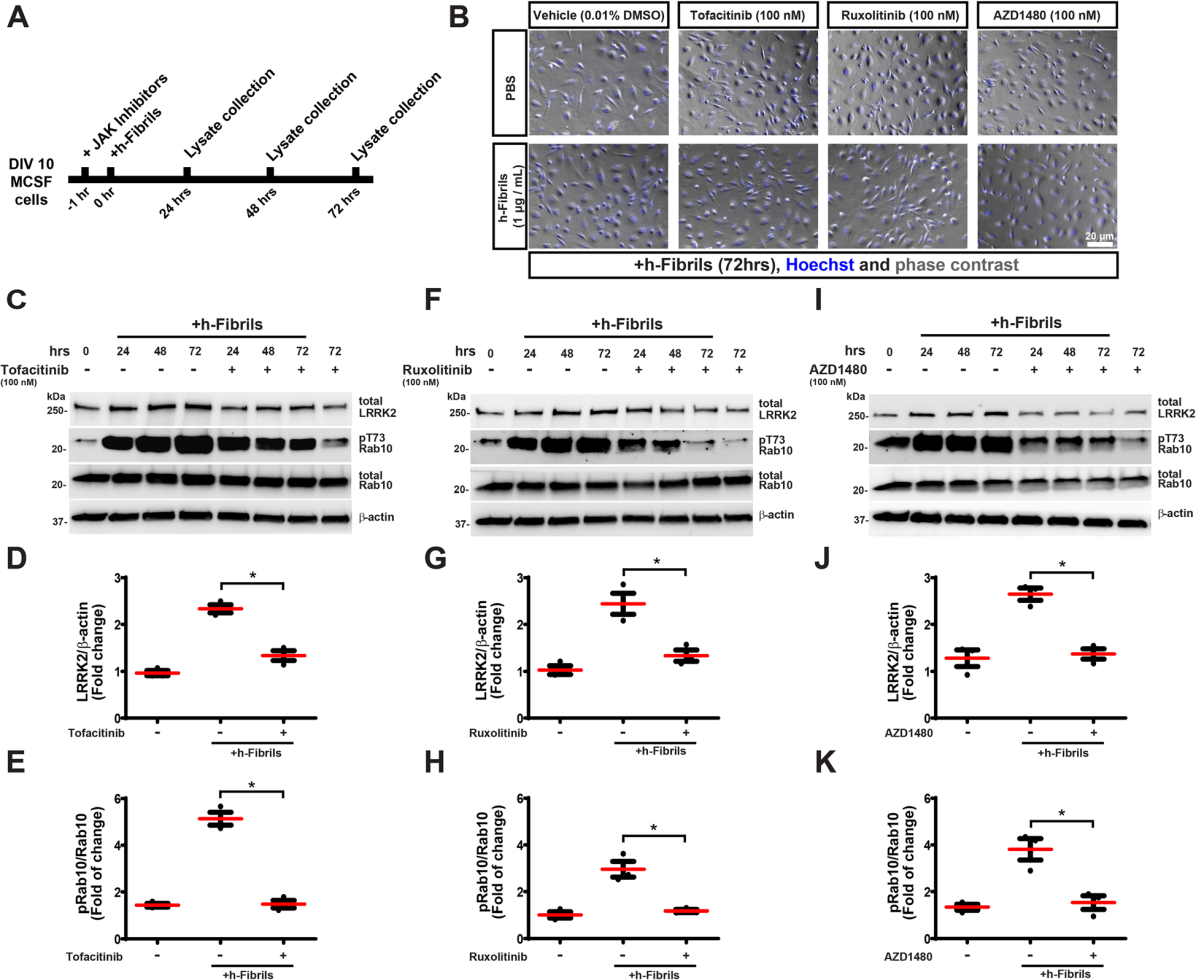
α-Synuclein fibrils induce LRRK2 activity through JAK-STAT signaling. **A** Timeline of JAK inhibitor pretreatment and human α-synuclein fibrils administration to human monocyte-derived macrophages (MCSF expanded). Cell lysates were collected at the time points indicated. **B** Representative phase-contrast images of the macrophages before lysate collection. Scale bar is 20 μm. **C** Representative immunoblots of lysates treated with human ɑ-synuclein fibrils (0.7 nM, or 1 μg・mL^− 1^) as indicated, with (+) or without (−) the JAK inhibitor Tofacitinib citrate (100 nM, JAK1/2/3). **D**-**E** Quantification from three independent experiments (two healthy males and one healthy female, age range 24 to 34 years old). of lysates analyzed at 72 h post-fibril exposure, with or without Tofacitinib as indicated. **F**-**H** Macrophages were treated with Ruxolitinib (100 nM, Jak1/2 selective) or **I**-**K** AZD1480 (100 nM, Jak2 selective), as indicated. Data are plotted as the mean (red bars) ± SEM. Significance was assessed by one-way ANOVA with Tukey’s post hoc test. *p < 0.05 for selected group comparisons

Past studies suggest that aggregated α-synuclein may stimulate TLR4 signaling pathways in innate immune cells [42, 43]. We and others have previously implicated LRRK2 in TLR4 signaling [19, 26, 44]. In human primary monocyte-derived macrophages, we found that the application of a potent TLR4 inhibitor failed to block α-synuclein fibril-induction of LRRK2 protein expression and Rab10 phosphorylation (Supplemental Fig. 2). To search for other pathways that might regulate LRRK2 induction, a comparative analysis of several transcriptomes of human primary monocyte-derived macrophages treated with α-synuclein fibrils highlighted components of JAK-STAT signaling including *STAT1* and *JAK2* mRNA together with *LRRK2* mRNA (Supplemental Fig. 3). We hypothesized that α-synuclein fibrils might stimulate the JAK-STAT pathway to promote LRRK2 expression. To test this possibility, we treated cultures with three structurally distinct JAK inhibitors 1 h prior to fibril administration and measured subsequent LRRK2 and Rab10 phosphorylation changes (Fig. 2). While ruxolitinib and AZD1480 primarily target JAK1/2 at low nanomolar concentrations [45, 46], tofacitinib-citrate primarily targets JAK2/3 [47, 48]. After fibril treatment, LRRK2 levels and activity were upregulated early and sustained consistently to 72 h post-fibril treatment in vehicle-only conditions (Fig. 2A-K). All three inhibitors effectively reduced α-synuclein fibril-induced LRRK2 expression levels and pT73-Rab10 levels, without apparent morphological changes to the cells (Fig. 2B). These results indicate that the upregulation of JAK-STAT signaling caused by fibril exposure is necessary for the upregulation of LRRK2 expression and subsequent Rab10 phosphorylation.

### Effect of innate immune cell differentiation on α-synuclein fibril induction of LRRK2

**Fig. 3:**
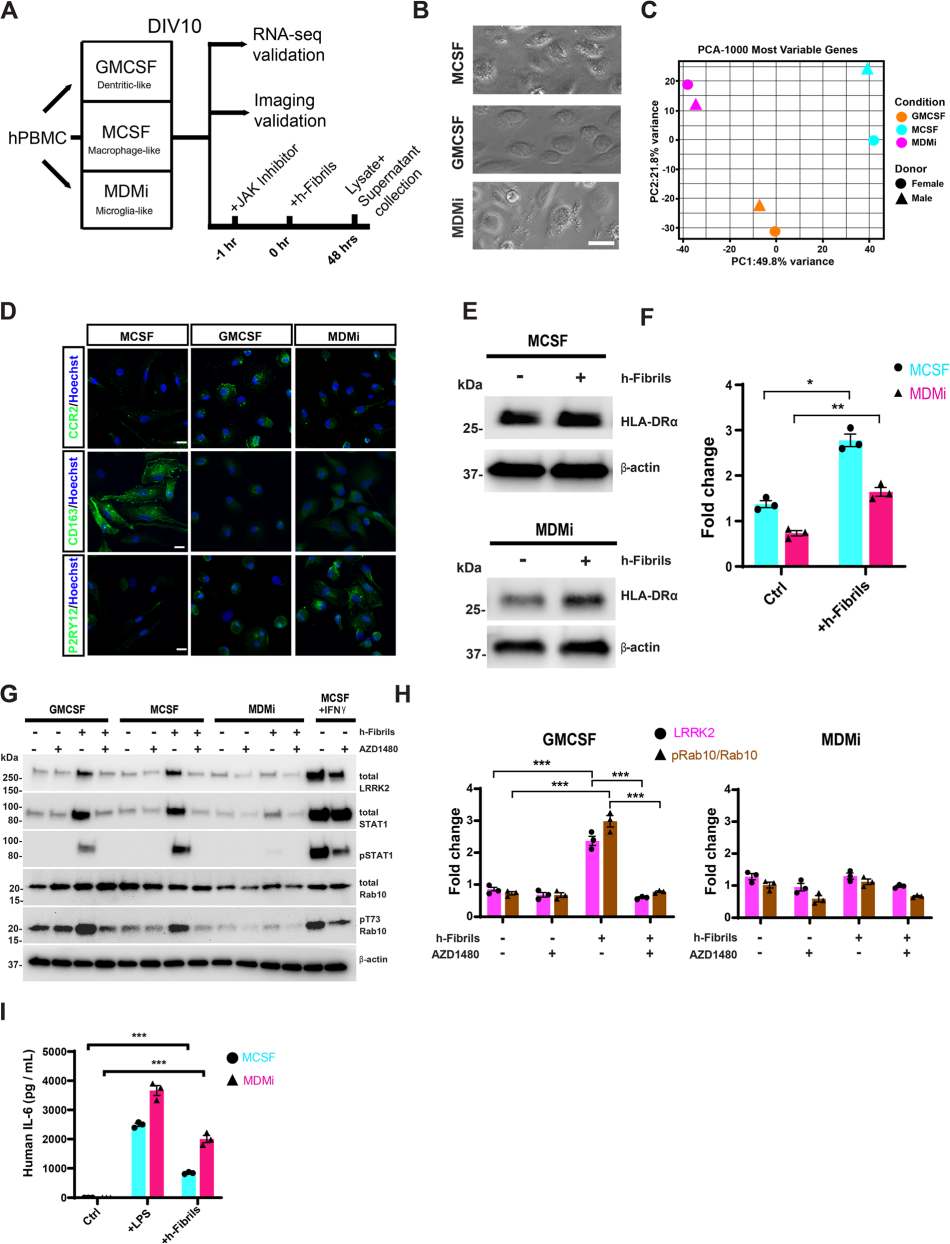
Microglia-like polarization suppresses α-synuclein fibril induction of LRRK2 expression and activity. **A** Flow chart of PBMC differentiation into three monocyte lineages, with validation (mRNA-seq and fluorescent imaging of canonical markers), and treatment timeline. The JAK inhibitor (AZD1480, 100 nM) was applied 1 h before human ɑ-synuclein fibrils (1 μg・mL^− 1^), and cell lysates were collected 48 h later. **B** Representative phase-contrast images of human monocytes differentiated to dendritic-like cells (GMCSF), macrophage-like cells (MCSF), and microglia-like cells (MDMi). **C** Principal component analysis of the resultant transcriptomes (circle: female, triangle: male, orange: GMCSF, cyan: MCSF, pink: MDMi). **D** Representative immunostaining of canonical markers for the different lineages (CD163 for macrophages MCSF; CCR2 for dendritic cells GMCSF; and P2RY12 for microglia cells MDMi). Quantification presented in Supplemental Fig. 4. Scale bars are 10 μm. **E** Representative immunoblots, and **F** quantification for HLA-DRα upregulation, 24 h after human α-synuclein fibril treatment in both macrophages and microglia-like (MDMi) cells. **G** Representative immunoblots of lysates from cells treated with or without human ɑ-synuclein fibrils (1 μg・mL^− 1^, 48 h), with or without the JAK2 inhibitor AZD1480 (100 nM). IFNγ (20 ng・mL^− 1^) treated macrophages (MCSF) were used as a positive control for LRRK2 upregulation and functional activation in the different cell lineages. **H** Quantification of total LRRK2 and the ratio of pT73-Rab10 to total Rab10 in dendritic (GMCSF) and microglia-like cells (MDMi). **I** From cell culture media, human IL-6 levels were measured by ELISA in untreated cells (control), cells treated with LPS (100 ng・mL^− 1^, 24 h), or human ɑ-synuclein fibrils (1 μg・mL^− 1^, 24 h). All data show mean ± SEM for n = 3 independent experiments, except for transcriptional analysis in panel C that includes a male and female pair (healthy controls, age range 24 to 34 years old). Significance is assessed by one-way ANOVA with Tukey’s post hoc test where *p < 0.01, **p < 0.01, ***p < 0.001

LRRK2 expression and function has been described in a variety of immune cells including neutrophils and monocytes [8, 49], and by immunohistochemistry in CD68+ macrophage subsets of unspecified origins that can accumulate in the rat brain in the context of LPS and rAAV-α-synuclein insults [15]. According to Ryan et al., naive human blood monocytes procured through negative selection can be polarized to functionally and transcriptionally resemble different types of innate immune cells including microglia-like (MDMi) cells [29]. CD14+/CD16+ monocytes were isolated from healthy volunteers and differentiated to resemble dendritic-like (GMCSF) cells, macrophage-like (MCSF) cells, and MDMi cells. Resultant cell populations differentiated for ten-days in culture (Fig. 3A) were morphologically similar in presentation (Fig. 3B) but transcriptionally distinct (Fig. 3C). Functional differentiation to these different states was characterized by CD163 expression that was high in MCSF cells, high CCR2 expression in GMCSF cells, and high P2RY12 in MDMi cells (Fig. 3D, with fluorescence intensities quantified in Supplemental Fig. 4). HLA-DR is a canonical pro-inflammatory surface marker robustly detected in PD brains [50], and fibrils stimulated HLA-DRα expression in both MCSF and MDMi cells (Fig. 3E, F). Similar to MCSF cells, GMCSF cells demonstrated upregulated LRRK2 and pT73-Rab10 levels after α-synuclein fibril exposures that could be completely mitigated by concurrent AZD1480 treatment (Fig. 3E, F). However, in MDMi cells, fibrils failed to stimulate LRRK2 or Rab10 phosphorylation. In contrast, MDMi cells robustly responded to the fibrils with IL-6 cytokine secretion to a similar (or slightly increased) extent as compared to MCSF macrophage cells (Fig. 3I). These results suggest that microglia have low levels of LRRK2 kinase activity in response to α-synuclein fibril exposures, in contrast to macrophages and dendritic-like cells that show strong induction of LRRK2 kinase activity in response to fibrils.

### α-synuclein fibrils recruit Lrrk2-monocytes to the brain

**Fig. 4:**
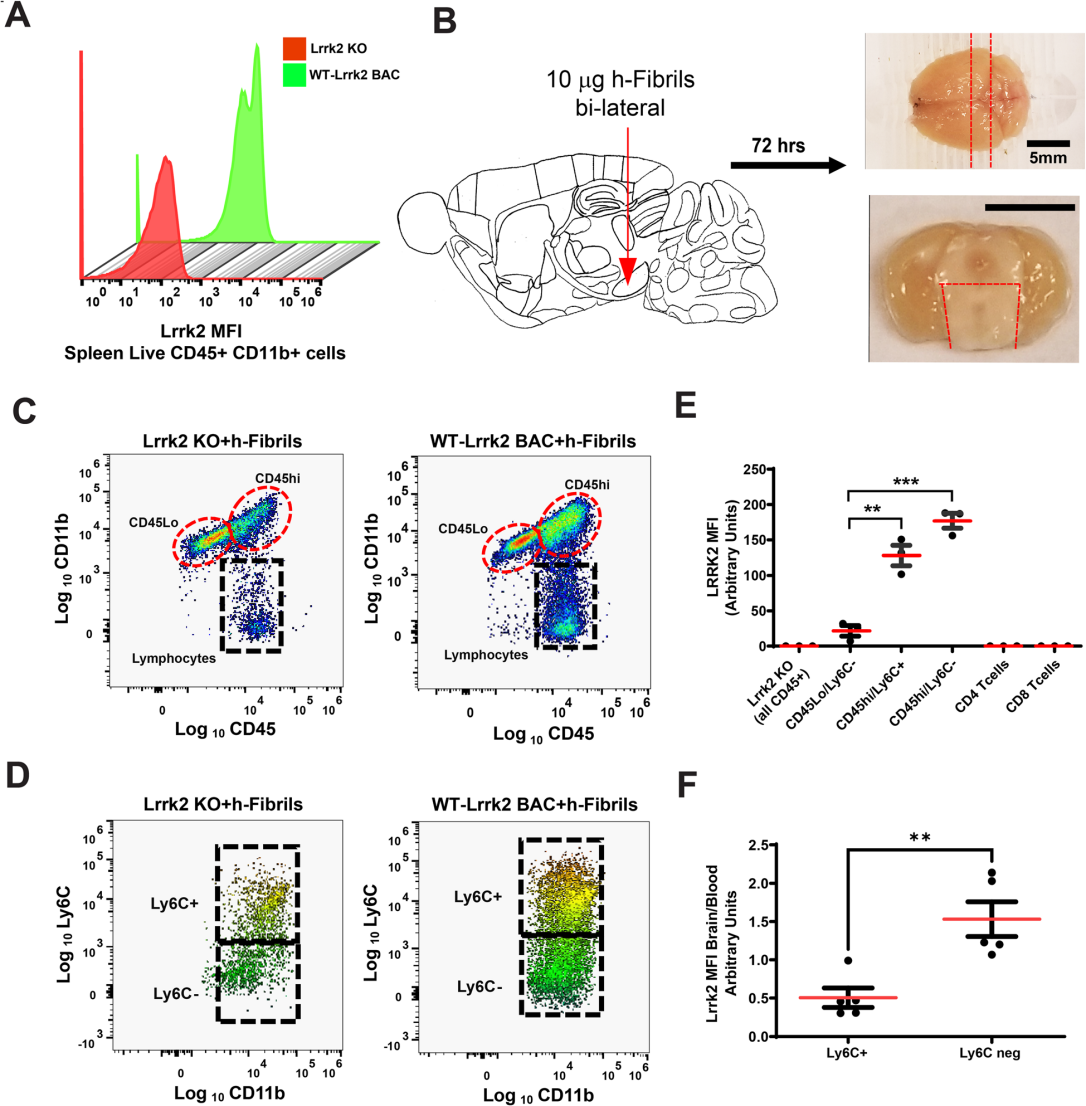
α-Synuclein fibrils induce Lrrk2 expression in classical and non-classical monocytes recruited to the mouse brain. **A** Analysis of median-fluorescence intensity (MFI) in spleen cells (live CD45+/CD11b+) isolated from naive Lrrk2 knockout (KO) and WT-Lrrk2 BAC mice with monoclonal antibody c41–2. **B** Sagittal aspect of a mouse brain showing the site of human α-synuclein fibril (10 μg, bilateral) injection (red arrow). After 72 h, the indicated midbrain region (boxed in red lines) was isolated and triturated for flow cytometry analysis. Scale bars shown for 5 mm. **C** Lrrk2 expression in immune cells (live CD45+/CD11b+) isolated from the midbrain. Representative cytographs with representative gates (dashed boxes) depicting CD45^Lo^, CD45^hi^, and lymphocyte populations. Extended gating strategy is detailed in Supplemental Fig. 6. **D** Ly6C+ (enriched in classical monocytes) and Ly6C- (enriched in non-classical monocytes) populations are shown from the CD45^hi^ gate. **E** Representative Lrrk2 MFI (relative to Lrrk2 KO) in different immune cell populations calculated (by subtracting MFI signals from Lrrk2 KO mice from each cell population) of brain cell suspensions from WT-Lrrk2 BAC mice as well as Lrrk2 KO mice. Data are plotted as the mean MFI (red bars) ± SEM for n = 3 mice (2 males and 1 female for WT-Lrrk2 BAC and 2 males for Lrrk2 KO), aged 2–3 months. **F** Lrrk2 MFI in CD45^hi^/CD11b+/Ly6C+ and CD45^hi^/CD11b+/Ly6C- cell populations were evaluated from midbrain cell suspensions and matched whole blood samples 72 h after human α-synuclein fibril (10 μg, bilateral midbrain) injection. The ratio of brain/blood Lrrk2 MFI, as measured via flow cytometry, is depicted. Data are plotted as the mean MFI (red bars) ± SEM for *n* = 5 mice in each group (3 males and 2 females for WT-Lrrk2 BAC mice aged 4 months. Significance was assessed by 2-sided t-test or one-way ANOVA with Tukey’s post hoc test, **p < 0.01, ***p < 0.001

In human brain tissue, activated antigen-presenting innate immune cells are often nearby neuronal α-synuclein inclusions [51]. Both α-synuclein over-expression or intracranial LPS injection causes the accumulation of CD68-positive macrophages in the brain that may originate from resident innate immune cells (e.g., microglia) or infiltrating bone-marrow-derived cells (e.g., monocytes) [15, 19, 52]. To track expression in immune cells isolated directly from the perfused mouse brain, we established a flow cytometry protocol with previously described Lrrk2-targeting rabbit monoclonal antibodies [25]. For optimization, initial Lrrk2 intracellular flow cytometry experiments were performed with spleen tissues to identify staining conditions compatible with Lrrk2 protein detection in monocytes, with the Lrrk2 knockout mouse strain as a control (Fig. 4A). We were unable to resolve specific endogenous Lrrk2 signals in WT (nTg) mice. In comparing mouse and human macrophages, we noticed mouse Lrrk2 was expressed at approximately one-fifth the level of human LRRK2 in the same number of cells cultured the same way, irrespective of IFNγ-induction or alternative polarization (Supplemental Fig. 5). Serendipitously, mouse cells overexpressing *Lrrk2* from a mouse BAC insertion had similar levels of Lrrk2 protein as human cells, controlled from the endogenous *Lrrk2* promoter (Supplemental Fig. 5). In the analysis of spleen cells from the WT-Lrrk2 BAC mice, we could successfully resolve Lrrk2-signals in monocyte-enriched CD45+/CD11b + populations (Fig. 4A).

To track possible Lrrk2-positive mouse immune cells in the brain after the intracranial injection of α-synuclein fibrils, we performed flow cytometry with midbrain tissue homogenates from the WT-Lrrk2 BAC mice side-by-side with Lrrk2 KO mice as controls. The injection strategy and tissue dissected for analysis by flow cytometry is illustrated in Fig. 4B. Cell populations were gated according to CD11b and CD45 positivity (Fig. 4C, with gating strategy provided in Supplemental Fig. 6). Both Ly6C+ (enriched in classical monocytes according to [53]) and Ly6C- (enriched in non-classical monocytes) cell populations emerged in the CD11b+/CD45^hi^ population with robust Lrrk2 expression (Fig. 4D, E). In contrast, the much larger CD11b+/CD45^Lo^/Ly6C- cell population (primarily microglia) had very low Lrrk2 or undetectable expression (Fig. 4E). The CD11b+/CD45^hi^ population was miniscule with saline-only 72 h post injection (Supplemental Fig. 7), consistent with recent reports in rats suggesting saline or monomeric α-synuclein injections do not result in the recruitment of peripheral immune cells to the brain [17]. No discernable signals were identified for Lrrk2 expression in the CD4 or CD8 T-cell populations that accumulated in the brain (Fig. 4E). After 72 h of intracranial injection of α-synuclein fibrils, Lrrk2 levels between infiltrated and peripheral (blood) populations of CD45^hi^/Ly6C+ and CD45^hi^/Ly6C+ were compared. The highest Lrrk2 levels we observed in this study localized to the CD45^hi^/Cd11b/Ly6C- cell compartment in the brain after fibril injection (Fig. 4E, F). Ly6C expression in CD45^hi^/Cd11b/Lrrk2^hi^ cells in the brain may reflect a dynamic loss of Ly6C expression once Ly6C^hi^ monocytes commensurate with monocyte to macrophage maturation known to occur in tissues [54, 55]. Alternatively, this could reflect a dynamic upregulation of Lrrk2 expression in non-classic CD45^hi^/Cd11b/Ly6C- cells that remain Ly6C negative with maturation, or some combination of both processes.

### Mutant Lrrk2 expression increases pro-inflammatory monocyte recruitment to the brain shortly after α-synuclein fibril injection

**Fig. 5:**
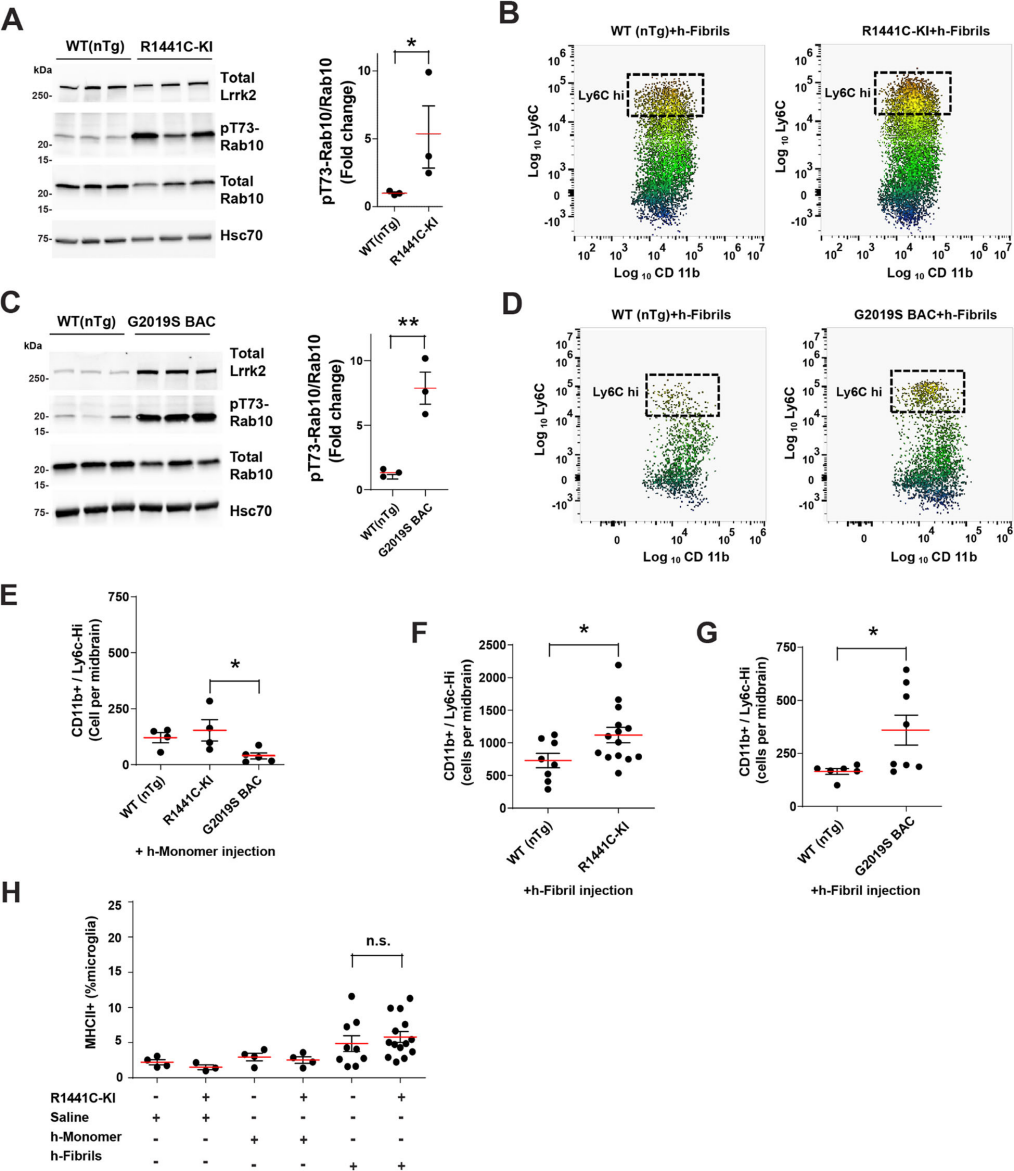
Increased α-synuclein fibril-induced infiltration of pro-inflammatory monocytes in *Lrrk2* knock-in R1441C and G2019S-BAC mice. **A** Representative immunoblots and quantification of Lrrk2 expression and Rab10 phosphorylation (pT73-Rab10 to total Rab10) in brain lysates from WT (nTg) mice and homozygous Lrrk2-R1441C knock-in (KI) mice, and **B** live CD45^hi^/CD11b+/Ly6C^hi^ midbrain cells isolated from a mononuclear-enriched Percoll gradient 3 days after injection (bilaterally) with 10 μg of human α-synuclein fibrils (gating strategy is presented in Supplemental Fig. 8). **C**, **D** Mice expressing G2019S-Lrrk2 with the mutation knocked into a mouse BAC spanning the *Lrrk2* gene were likewise analyzed together with littermate WT (nTg) controls. **E** Quantification of CD45^hi^/CD11b+/Ly6C^hi^ (enriched in pro-inflammatory classical monocytes) cells isolated from the brains of WT (nTg), Lrrk2-R1441C KI and G2019S BAC mice 72 h after the injection of 10 μg (bilaterally) of monomeric human α-synuclein. **F** In contrast to monomeric protein injections, the injection of fibrils (10 μg bilateral, 72 h wait) results in the robust recruitment of CD45^hi^/CD11b+/Ly6C^hi^ cells in the mouse brain (PBS perfused and then analyzed by flow cytometry, see Methods) that is further increased in R1441C-KI mice and **G** G2019S-BAC mice. Microglia cell numbers after fibril treatment between strains are not different between groups (see Supplemental Fig. 8). **H** Percentage of activated microglia (CD45^lo^/CD11b+/MHCII+) in WT (nTg) and R1441C-KI mice injected with saline, human α-synuclein monomers, or fibrils (10 μg bilateral) is shown in the graph. In each panel, each dot represents the analysis of an individual mouse with 13 mice (3 males and 10 females) analyzed in **E**, 22 mice (9 males and 13 females) analyzed for **F**, 14 mice (5 males and 9 females) analyzed for **G**, and 37 mice (13 males and 24 females) analyzed for **H**. All mice were aged 2–3 months at the time of the analysis. Data are plotted as the mean (red bars) ± SEM. Significance is determined by unpaired *t*-tests *p < 0.05, **p < 0.01

Previously, we found that intracranial injections of α-synuclein fibrils into rat substantia nigra, but not monomeric protein or saline control, causes microglial activation and the upregulation of MHCII expression, as well as the recruitment of different proinflammatory immune cells to the brain [17]. Given our current observation that Lrrk2 expression appears inducible with α-synuclein fibrils in the monocyte cell compartment, and our past work that suggests LRRK2 might control macrophage chemotaxis through Rab10 phosphorylation [9, 10], we sought to determine whether pathogenic LRRK2 mutations that upregulate LRRK2 kinase activity might affect the recruitment of monocytes to the brain in response to α-synuclein fibrils. We employed recently developed congenic (C57BL/6 J) R1441C-Lrrk2 knock-in (KI) mice as well as G2019S-BAC mice that show upregulated Lrrk2 expression and activity for in vivo chemotaxis experiments. In brain lysates from homozygous R1441C-KI mice, as well as G2019S-Lrrk2 BAC mice, we detected an increase of the Lrrk2 substrate pT73-Rab10 compared to their WT(nTg) littermate controls in naive animals (Fig. 5A, C). To measure immune cell responses in the brain after exposure to α-synuclein fibrils, we performed flow cytometry analysis with tissue dissected according to Fig. 4B, 72 h after bi-lateral fibril injection. Gating strategies for these cohorts of mice are described in Supplemental Fig. 8. To account for the possible effects of surgery and the injection of saline, as well as the non-specific effects of the recombinant α-synuclein protein, we first analyzed immune cell responses 72 h after monomer protein injection. With monomer only exposures, no differences were noted in the numbers of monocytes (CD11b+/Ly6G−/Ly6C^hi^) in the midbrain between WT(nTg) mice and R1441C-KI mice, though a slight suppression of the number of Ly6C^hi^ monocytes was noted in G2019S-Lrrk2 BAC mice midbrain homogenates (Fig. 5E). Thus, both lines of mutant Lrrk2 mice did not display basal differences in the brain with respect to monocyte numbers. However, with α-synuclein fibril injections, we observed an increase in the number of CD11b+/Ly6G−/Ly6C^hi^ monocytes (i.e., pro-inflammatory infiltrating) in both R1441C-Lrrk2 KI (Fig. 5B) as well as G2019S-Lrrk2 BAC (Fig. 5D) mice when compared to their corresponding littermate WT(nTg) controls (Fig. 5F, G). No differences in the numbers of the more abundant microglia cell population was noted between the different strains (Supplemental Fig. 8), nor differences at this time point in the numbers of MHCII-positive activated microglia in the R1441C-Lrrk2 KI mice treated with monomers or fibrils (Fig. 5H). These results suggest mutated LRRK2 may exacerbate the recruitment of pro-inflammatory monocyte-derived macrophages to the brain in response to pathogenic α-synuclein.

### Lrrk2 kinase inhibition blocks monocyte-derived macrophage chemotactic responses to α-synuclein fibrils

**Fig. 6:**
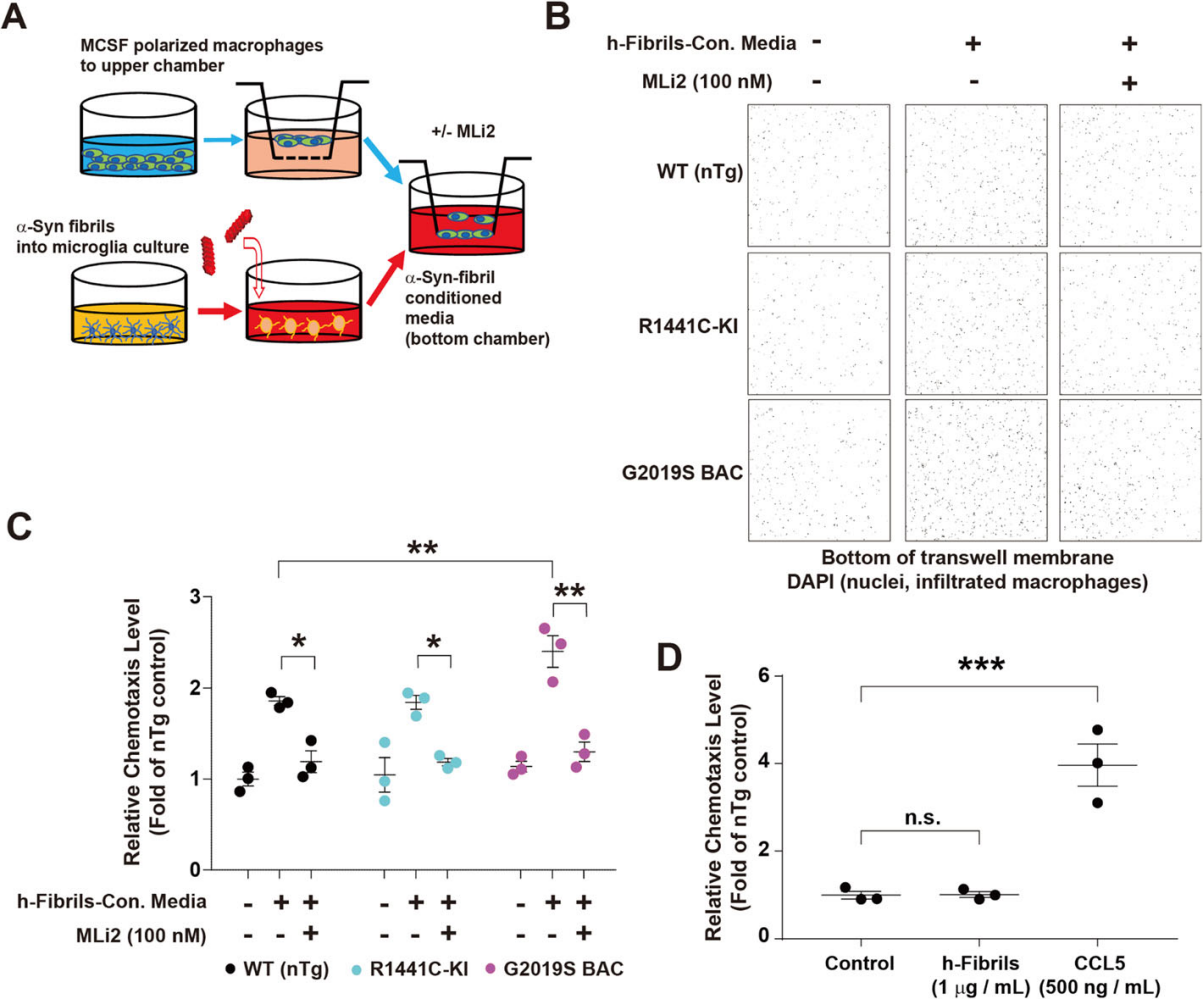
Lrrk2 kinase inhibition attenuates chemotactic responses to α-synuclein fibrils. **A** In the experimental paradigm, mouse BV-2 microglial cells were treated for 24 h with human α-synuclein fibrils (+) or monomer (−) control (both at 1 μg・mL^− 1^), and cell culture media was applied (1/10th volume) to the bottom chamber in Boyden transwell assays. Chemotaxis of bone-marrow derived macrophages (MCSF) cells from the indicated mouse strain were compared, with or without the Lrrk2 small molecule inhibitor MLi2 (100 nM). **B** After 2 h, cells that failed to migrate into the transwell membrane were removed with a cotton swab. Migrated cells were stained with DAPI, counted across the filter, and representative images from three independent experiments from three mice (male) are shown, and **C** relative chemotaxis calculated as a fold of the number of WT(nTg) cells from three different experiments. Each dot represents one independent experiment. **D** Human α-synuclein fibrils (1 μg・mL^− 1^) were added into the bottom chamber of Boyden transwell assays. Chemotaxis of bone-marrow derived macrophages cells were counted after 2 h. CCL5 (500 ng・mL^− 1^) was used as the positive control. Data are group means ± SEM. Significance was assessed by one-way ANOVA with Tukey’s post-hoc test where *p < 0.05, **p < 0.01, and ***P < 0.001

Previously we and others found that α-synuclein fibrils, but not monomeric protein, elicits the strong secretion of a number of murine chemokines and cytokines from microglia-like BV-2 cells, a mouse cell line with microglia features, including Ccl5 secretion known to stimulate monocyte chemotaxis [17, 56]. LRRK2 kinase inhibition may attenuate chemotaxis in monocyte-derived macrophages, possibly through blocking Rab10 phosphorylation that boosts Ccl5-induced AKT activation [9]. We tested whether LRRK2 kinase inhibition might likewise have a direct effect on chemotactic responses in the context of WT or mutant Lrrk2 (R1441C-Lrrk2 KI or G2019S-Lrrk2 BAC) expression in mouse monocyte-derived macrophages (Fig. 6A). The application of conditioned media from α-synuclein fibril-treated BV-2 cell cultures led to a ~ 2-fold increase in the number of macrophages migrating into the Boyden chamber matrix over 2 h (Fig. 6B, C). The addition of the LRRK2 kinase inhibitor MLi2 (100 nM) together with the α-synuclein fibril conditioned media in the Boyden chamber blocked the increases in chemotaxis. In these cells, the effects of mutant Lrrk2 expression were less apparent, with the G2019S-Lrrk2 BAC cells showing increased numbers of migration into the matrix in response to the α-synuclein fibril conditioned media (Fig. 6C). The α-synuclein fibrils are not themselves chemoattractants, as exposures of equivalent concentrations of fibrils used to treat the BV-2 cells to the lower half of the Boyden chambers did not stimulate chemotaxis (Fig. 6D). Together, these results support a link between LRRK2 kinase activity and α-synuclein fibril-induced chemotactic responses.

## Discussion

**Fig. 7:**
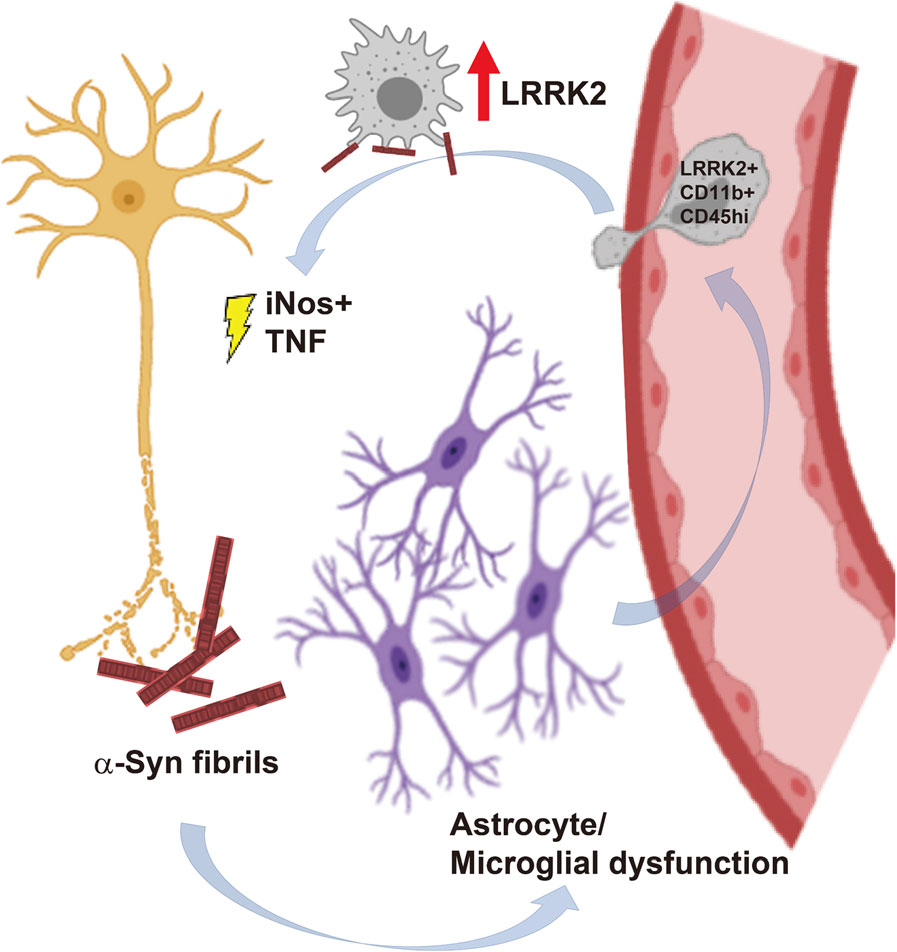
Model for possible LRRK2 interaction with ɑ-synuclein fibrils in neuroinflammation and neurodegeneration. Neuronal terminal damage, caused in part by the fibrillization and aggregation of ɑ-synuclein, may cause microglia and astrocyte responses that deleteriously affect the blood-brain barrier, the secretion of chemokine gradients, or possibly the release of aggregated ɑ-synuclein. Monocyte extravasation may occur with concomitant sensing of ɑ-synuclein fibrils that elicit LRRK2 expression to facilitate chemotaxis into parenchymal tissues. Pro-inflammatory monocytes may control local production of damaging cytokines and oxidative responses to cause further neuronal damage in a feed-forward progressive pathway

In this study, we found that LRRK2 induction and activity in response to α-synuclein fibrils is robust in monocyte-derived macrophages but largely absent in resident brain microglia in mice. LRRK2 induction via fibrilized α-synuclein in cultured cells depends on JAK-STAT signaling as well as intrinsic LRRK2 kinase activity. These relationships appear conserved between mouse and human cultured immune cells, and between mouse and human strains of α-synuclein fibrils, although lower levels of Lrrk2 are observed in mouse cells as compared to human cells. The mechanisms of LRRK2 induction observed here are largely consistent with prior studies that have shown that LRRK2 synergizes with interferon responses in iPSC-derived cells, and promotes LPS signaling in R1441G-transgenic mice [20, 26]. These results are also potentially consistent with previous studies suggesting LRRK2 kinase activity affects vesicle turnover and traffic in the endolysosomal system to strengthen chemokine signaling, possibly through a Rab10 and CCR5 dependent pathway [10]. Taken together, we hypothesize that LRRK2 drives neuroinflammatory responses in part by enhancing the recruitment of pro-inflammatory monocytes from the periphery. These processes may synergize with LRRK2 effects in the periphery that may also amplify pro-inflammatory responses to affect cells in the brain [26]. Following this hypothesis, future studies that specifically repress or activate LRRK2 only in the monocyte cell compartment in the context of disease may provide critical additional insights. A hypothesized model is proposed in Fig. 7, where chemoattractants from microglia, astrocytes, and other cells responsive to α-synuclein may stimulate the recruitment of damaging pro-inflammatory LRRK2-positive immune cells.

We found inhibition of JAK-STAT signaling to be a potent means of blocking α-synuclein fibril induction of LRRK2 expression and activity. One of the JAK1/2 inhibitors used here, AZD1480, also provides protection against rAAV2-α-synuclein induced neuroinflammation and dopaminergic neurodegeneration in the brain [33]. As LRRK2 inhibitors also may provide protection in a similar rAAV2-α-synuclein model [57], we hypothesize that some of the neuroprotection afforded by AZD1480 may be through blocking LRRK2 induction in pro-inflammatory monocytes. Direct LRRK2 function and synergism in JAK-STAT signaling has yet to be fully clarified but may involve endocytosis, signaling endosome dynamics, and vesicle recycling. In intracellular analysis, the numbers of phospho-Rab10 vesicles were not increased with α-synuclein-fibril stimulation. Instead, the amount of phospho-Rab10 on the vesicles was increased. These results may be in line with recent observations that show that phospho-Rab10 vesicles fail to recycle and thus persist in the cytosol, thereby strengthening signal transduction [9]. As opposed to more ubiquitous Rab substrates, LRRK2 is both inducible and highly restricted in expression across different immune cell subtypes (Supplemental Fig. 9). This may explain in part why the effect of LRRK2 on different immunological outcomes may be highly contextual and refined.

Based on past studies that demonstrate robust LRRK2 effects on microglial responses both in cell culture and in mouse models [19–22], it has been widely presumed (by us and others) that the intrinsic action of LRRK2 in microglia must drive immunological phenotypes. Yet, emergent single-cell and transcriptome wide sequencing databases have failed to identify appreciable *Lrrk2* mRNA in mouse cells assigned to microglia lineages or brain-resident macrophage populations (Supplemental Fig. 9). Microglial cells in culture are known to acquire macrophage-like phenotypes distinct from authentic microglia in the mouse brain [58]. In a comparative analysis of cultured cells, we found that the cytokines and chemokines used to induce microglial-like states also suppress LRRK2 activation, but do not repress a robust response to α-synuclein fibrils. How closely these artificially-directed cells mimic those that might occur in the brain and periphery in disease is not clear. Further, we cannot exclude the possibility of disease-state specific immune cell populations (e.g., rare T-cell subpopulations) that might rely on LRRK2 for appropriate signaling in disease. Future studies will be required to pinpoint how brain-resident pools of microglia might suppress Lrrk2 expression compared to monocyte-derived macrophage cells. We expect high-resolution deep single-nucleus sequencing efforts, particularly in patient cells and brain tissue samples, to help pinpoint brain-engrafting macrophages and rare subpopulations of cells that may be physiologically important.

In rodent models of PD, peripheral immune cells interact with resident immune cells in diseased tissue [59]. Past studies implicate peripheral and systemic immunological states that can influence α-synuclein fibril-induced phenotypes in the brain [60]. In later time points (months) after fibril injection, but before dopaminergic neurodegeneration, recent reports have highlighted persistent infiltration of B cells, T-cells, and natural killer cells in the brain [16]. Increases in leukocyte subsets in the spleen and lymph nodes were also detected, although minimal changes in blood were noticed ~ 5 months’ post-fibril injections. Based on our results, we would predict that early enhanced recruitment of pro-inflammatory cells to the brain might further exacerbate damaging systemic responses also mediated by LRRK2, with respect to dopaminergic neurodegeneration and the loss of other vulnerable neurons [26].

Further highlighting systemic responses in PD, two past reports suggest LRRK2 protein upregulation in human CD16+ blood monocytes using flow-cytometry [7, 8]. Notably, the LRRK2 antibody utilized here (rabbit monoclonal [MJFF2 (c41–2)]) has similar affinity to both mouse and human LRRK2 in recognizing a conserved epitope [8, 25, 61]. Based on our results and those of others, we predict that upregulated LRRK2 expression and activity in blood monocytes may be associated with increased pro-inflammatory monocyte responses in the brain (Fig. 7). Whether or not these cells contribute to damaging cytokine and oxidative responses in the brain remains to be determined in future studies. A potential therapeutic strategy to address the effects of increased LRRK2 expression and activity in monocytes may be to utilize agents that mitigate monocyte infiltration to the brain. However, an alternative hypothesis for LRRK2 function in monocytes in disease may include LRRK2 regulation of circulating factors that may stimulate effects in the brain [26]. Further, such systemic effects may synergize with local immune cell recruitment to the brain in disease. Additional studies that help untangle these relationships could have important consequences for successful therapeutic approaches to mitigate LRRK2 activity.

In targeting pathways important for mounting immunological responses to different types of challenges, there could be adverse consequences with blocking LRRK2 in innate immune cells. Some of these issues have been discussed by others recently and in depth [44, 62]. Future studies utilizing chemokine-receptor antagonists and other therapeutic candidates in the context of over-active mutant LRRK2 and α-synuclein fibril-induced neurodegeneration may be informative. In macrophages, we recently identified an interaction between LRRK2 kinase activity and CCR5 traffic via Rab10 phosphorylation [9]. Although there is complicated redundancy in chemokine signaling pathways in vivo and more interactions that remain to be identified with respect to LRRK2 signaling, the inhibition of CCR5 on monocytes that express high LRRK2 levels may present an opportunity to mitigate the effects of mutant LRRK2 in the immune system.

While the effects of LRRK2 kinase inhibition on chemotaxis were unambiguous in cultured cells, we were unable to perform the same LRRK2 kinase inhibitor experiment in living mice with a full mammalian immune system. In rats, we and others have described acceptable pharmacokinetics and brain penetration with standard oral delivery for several structurally distinct LRRK2 inhibitors known to block Rab phosphorylation [35, 57, 63–65]. But in mice, we are not aware of a selective and brain penetrant LRRK2 inhibitor with a half-life greater than one-hour in oral dosing that might produce long-duration inhibition. In-diet dosing regimens have been successfully utilized in mice, harnessing the normal feeding behavior in mice to counteract the rapid turn-over and micromolar concentrations of drugs associated with off-target binding in oral, IP, or IV dosing [40]. However, feeding behavior is disrupted in many animal models of disease including the surgical procedure of intracranial injection of fibrils used in this study. In addition, even in the context of non-disrupted in-diet dosing in healthy mice, mutant G2019S LRRK2 appears resistant to full kinase inhibition with several inhibitor classes [35, 66]. We further avoided observations in the global LRRK2 knockout model where compensatory mechanisms and lower white-blood cell counts may confound interpretations [67]. We predict that future studies that use genetic or transplantation methodologies to evaluate LRRK2 function in immune cells, as well as the evaluation of immune cell status in LRRK2 mutation carrier brain tissue, will be more informative in interpreting the function of LRRK2 in monocytes recruited to the brain.

## Conclusions

Here we describe two new potential roles for LRRK2 function in monocyte-derived macrophages reacting to aggregated α-synuclein. First, LRRK2 may facilitate initial chemotaxis in cells that express high LRRK2 levels, especially pro-inflammatory monocytes, that bring the cells into the brain parenchyma in response to the accumulation of aggregated α-synuclein. Second, once exposed to aggregated α-synuclein, JAK-STAT signaling may direct LRRK2 expression and kinase activity to bolster pro-inflammatory responses. Considering the role of LRRK2 kinase activity in enhancing ongoing interferon and NFATc2 signaling associated with damaging pro-inflammatory responses and chronic inflammation [20, 21], this process may have an overall detrimental effect on the survival of nearby vulnerable neurons. As LRRK2-targeted inhibitors enter into efficacy trials in both *LRRK2* mutation carriers and idiopathic disease, our results place further emphasis on peripheral immune cells positive for LRRK2 expression in the disease process. We anticipate that part of the therapeutic benefit of LRRK2 inhibition, if realized in the clinic, may be through reducing pro-inflammatory responses of brain-engrafting monocyte-derived macrophages that accumulate over the course of the disease.

## Supplementary Information

**Additional file 1.**

**Additional file 2.**

**Additional file 3.**

## Data Availability

The datasets used and/or analysed during the current study are available from the corresponding author on reasonable request.

## Abbreviations

LRRK2: Human leucine-rich repeat kinase 2
Lrrk2: Mouse leucine-rich repeat kinase 2
BMDM: Mouse bone marrow-derived macrophage
GMCSF: Granulocyte macrophage colony stimulating factor
LPS: Lipopolysaccharide
JAK: Janus kinase
MCSF: Macrophage colony stimulating factor
MDMi: Monocyte-derived microglia-like cells
PBMCs: Peripheral blood mononuclear cells
TLR: Toll-like receptor
STAT: Signal transducer and activator of transcription
