# Supplementary material for: Pathological α-synuclein recruits LRRK2 expressing pro-inflammatory monocytes to the brain
Source: Mol Neurodegener. 2022 Jan 10;17:7. doi: 10.1186/s13024-021-00509-5 (PMC8751347; doi:10.1186/s13024-021-00509-5)
Supplement: Supplementary file 1 — Additional file 1. [file 13024_2021_509_MOESM1_ESM.pdf]

Pathological α-Synuclein induces LRRK2 expression and activity in pro-inflammatory monocytes recruited to the brain

Enquan Xu, PhD[1,2], Ravindra Boddu, PhD[1,2], Hisham A. Abdelmotilib[3], Arpine Sokratian[1,2], Kaela Kelly, PhD[1,2], Zhiyong Liu, PhD[1,2], Nicole Bryant[1,2], Sidhanth Chandra[4], Samantha M. Carlisle, PhD[5,6], Elliot J. Lefkowitz, PhD[7], Ashley S. Harms, PhD[8], Etty N. Benveniste, PhD[9], Talene A. Yacoubian, MD, PhD[8], Laura A. Volpicelli-Daley, PhD[8], David G. Standaert, MD, PhD[8], Andrew B. West, PhD[1,2*]

Author affiliations:

[1]Duke Center for Neurodegeneration Research, Duke University, Durham, NC 27710, USA
[2]Department of Pharmacology and Cancer Biology, Duke University, Durham, NC 27710, USA
[3]Department of Neurology, University of Iowa, Iowa City, IA, USA
[4]Medical Scientist Training Program, Northwestern University Feinberg School of Medicine, Chicago, Illinois, 60611, USA
[5]Center for Clinical and Translational Science, University of Alabama at Birmingham, Birmingham, AL, 35294, USA
[6]Department of Chemistry and Biochemistry, New Mexico State University, Las Cruces, NM, 88003, USA
[7]Department of Microbiology, University of Alabama at Birmingham, Birmingham, AL, 35294, USA
[8]Center for Neurodegeneration and Experimental Therapeutics, Department of Neurology, University of Alabama at Birmingham, Birmingham, AL 35216, USA
[9]Department of Cell, Developmental and Integrative Biology, University of Alabama at Birmingham, Birmingham, AL 35294, USA

*Correspondence to: Andrew West, 3 Genome Court, Durham, NC 27710

andrew.west@duke.edu

Supplemental Figures

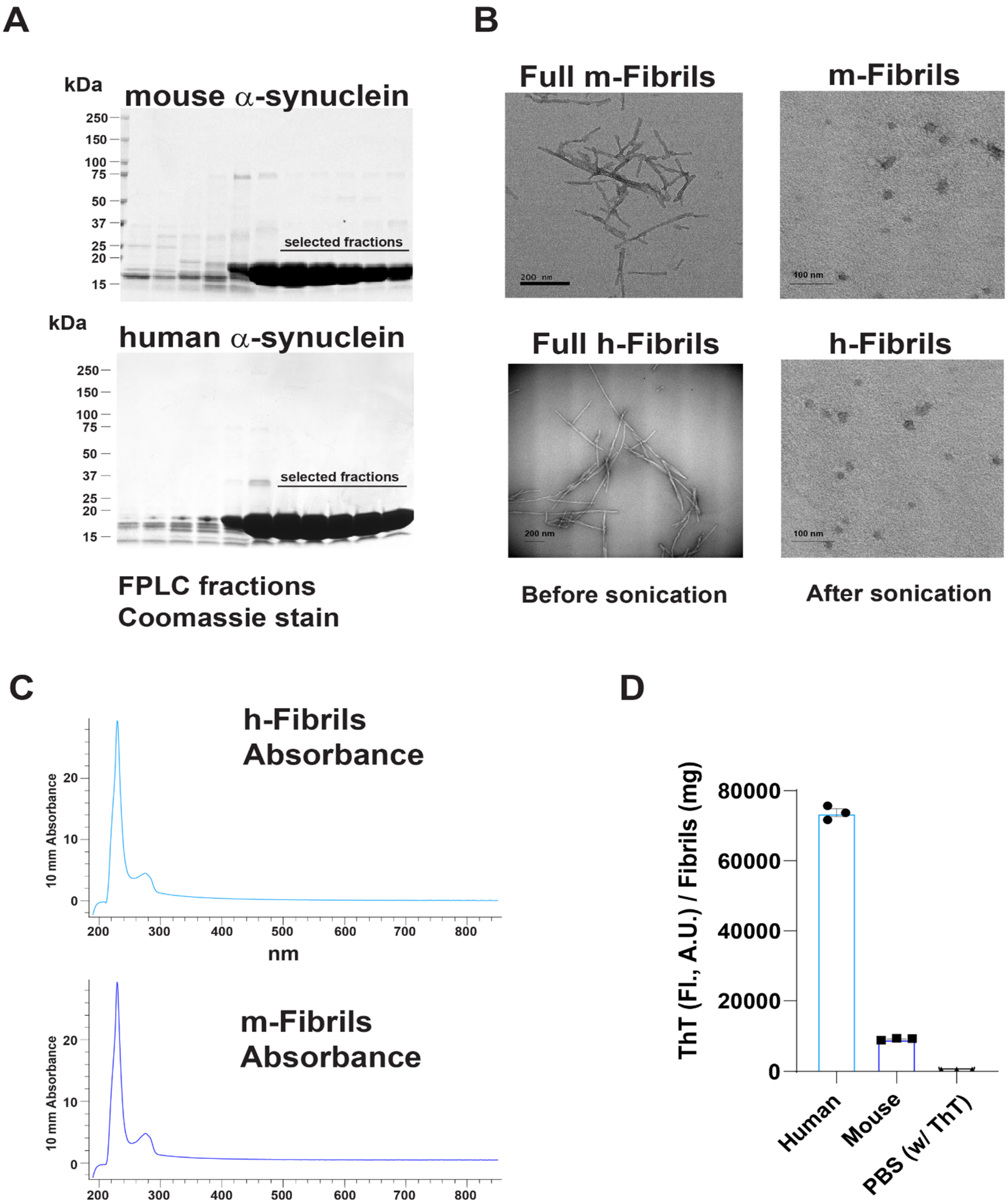

Supplemental Figure 1. Additional representative quality control of mouse and human α-synuclein fibrils. **(A)** Coomassie blue staining images of different fractions of FPLC in mouse and human α-synuclein monomer purifications. Selected fractions as indicated were pooled for

further processing (see Methods). **(B)** Electron microscopy images of mouse and human α-synuclein fibrils representative of before (fibrils with full length) and after sonication. Scale bars for full fibrils are 200 nm and scale bars for images of fibrils after sonication are 100 nm. **(C)** Spectral absorbance of mouse and human α-synuclein fibril preparations. **(D)** Thioflavin-T (ThT) binding fluorescence signals of mouse and human α-synuclein fibrils. Processed human fibrils demonstrate higher ThT fluorescence and binding as compared to similar mouse fibrils.

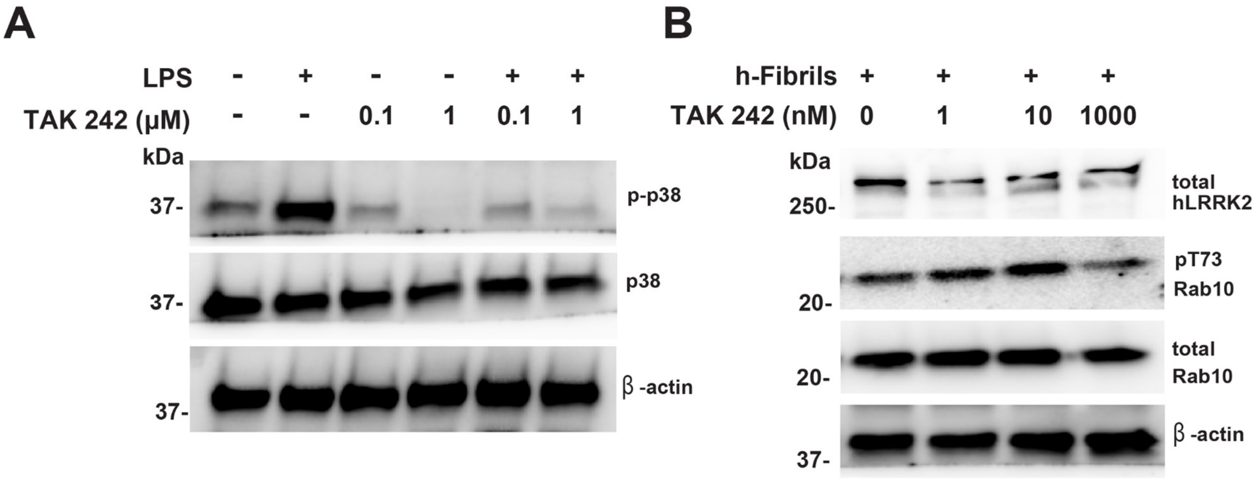

Supplemental Figure 2. TLR4-inhibitor TAK-242 does not attenuate α-synuclein fibril induction of LRRK2 expression and Rab10 phosphorylation. (A) Human primary monocyte-derived macrophages (MDMs) were pre-treated with TAK 242 (Resatorvid, typical IC_{50} in cells ~10 nM) [1] at the indicated concentrations, 1 hr pre-treated, before LPS (100 ng ▪ mL^{-1}) exposure. After 6 hrs of LPS treatment, cell lysates were analyzed by immunoblotting with p38 and phospho-p38 (downstream of TLR4 signaling pathway) to validate the efficacy of TAK-242 in the macrophages. **(B)** Macrophages were pre-treated with TAK-242 at the indicated concentrations 1 hr before application of α-synuclein fibrils (1 µg ▪ mL^{-1}). After incubation with fibrils for an additional 48 hrs, cell lysates were collected and analyzed by immunoblotting for LRRK2, phospho-Rab10, and total Rab10. Results are representative of three experiments.

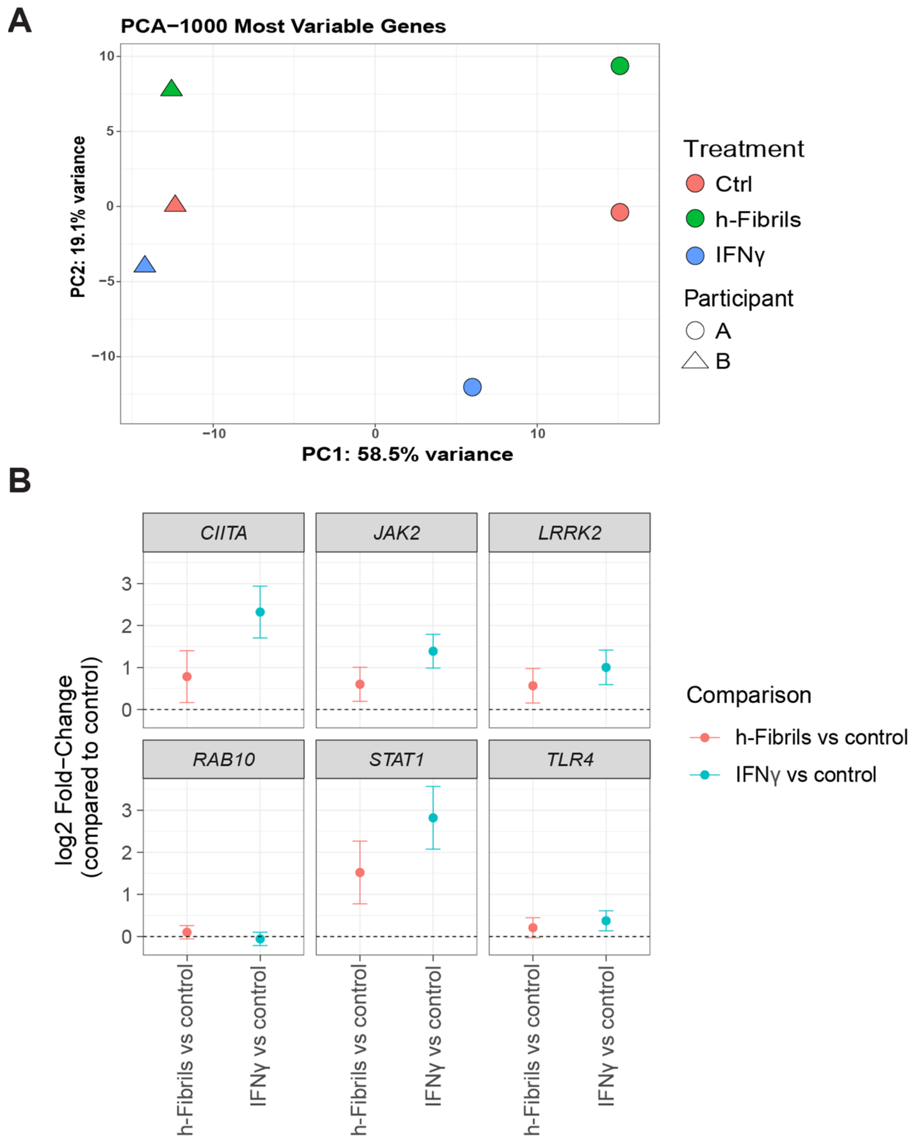

Supplemental Figure 3. Transcriptomic analysis of α-synuclein fibril treated human macrophages in comparison to interferon-γ treated cells. (A) Principal component analysis, and **(B)** candidate transcripts between the cell culture conditions from sequencing analysis. Human macrophages were treated with α-synuclein fibrils (1 μg ⋅ mL^{-1}) for 24 hrs, or IFNγ (20 ng ⋅ mL^{-1}) for 6 hrs, prior to Trizol RNA isolation.

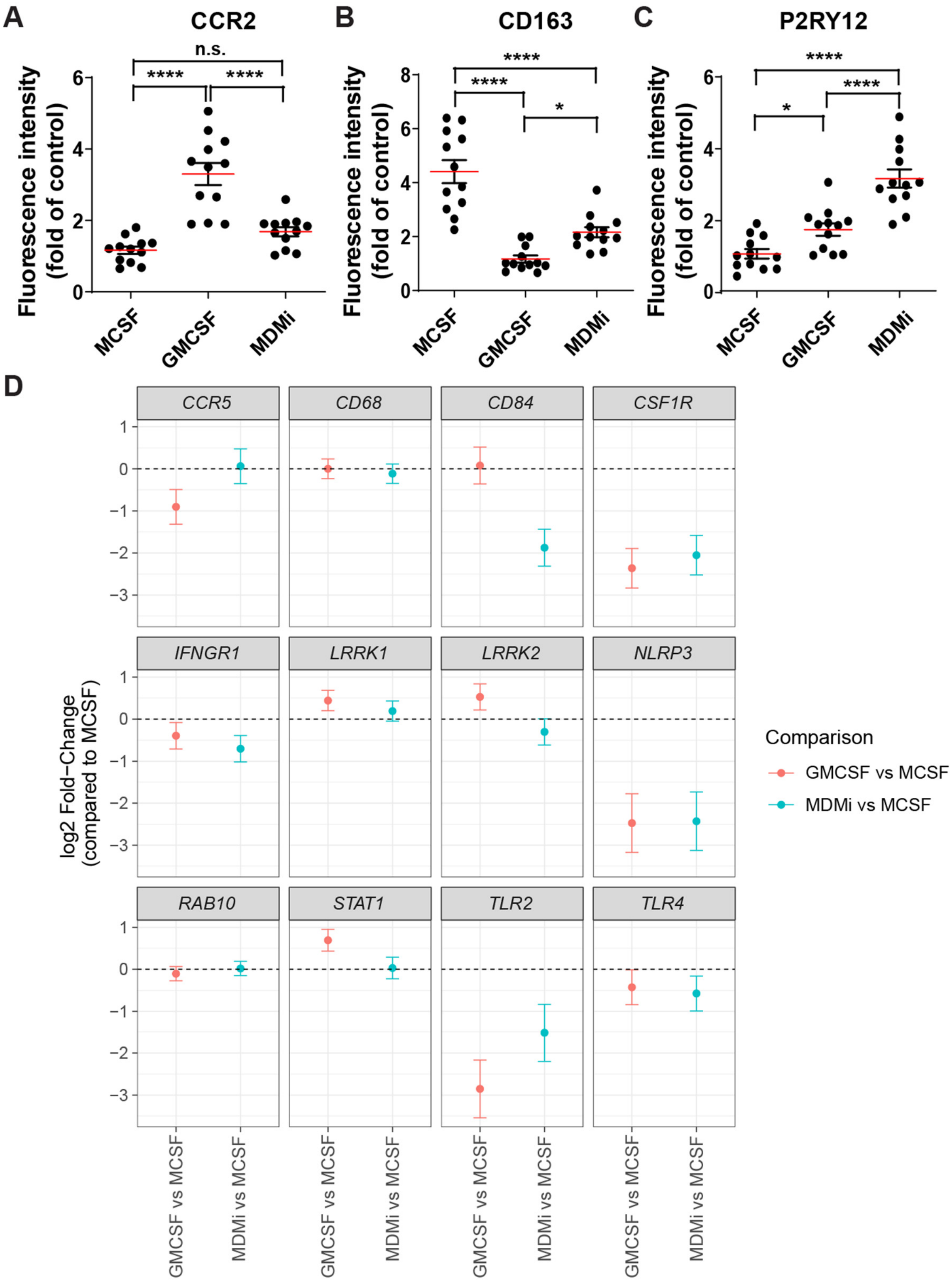

Supplemental Figure 4. Expression levels of selected markers and genes in monocytes polarized to different states. Human monocytes were polarized to GMCSF cells (dendritic-like monocytes): human GMCSF (5 ng・mL^{-1}) for 5 days, MCSF cells (monocyte-derived

macrophages), human MCSF (20 ng ▪ mL^{-1}) for 5 days, or MDMi cells (monocyte-derived microglia-like), GMCSF (10 ng ▪ mL^{-1}), MCSF (10 ng ▪ mL^{-1}), NGF-β (10 ng ▪ mL^{-1}), CCL2 (100 ng ▪ mL^{-1}), and IL-34 (100 ng ▪ mL^{-1}) for 10 days. (**A-C)** Quantification of the relative immunofluorescence signal of the indicated marker, with each dot representing an independent image from three different experiments in total. Relative fluorescence intensities are plotted as the mean value of MCSF treated cells. **(D)** Relative fold changes are indicated, as assessed by sequencing analysis. Red lines in graphs show group means with error bars depicting ± SEM. Significance is assessed by one-way ANOVA with Tukey's post hoc test, *p<0.05 and ****p < 0.001.

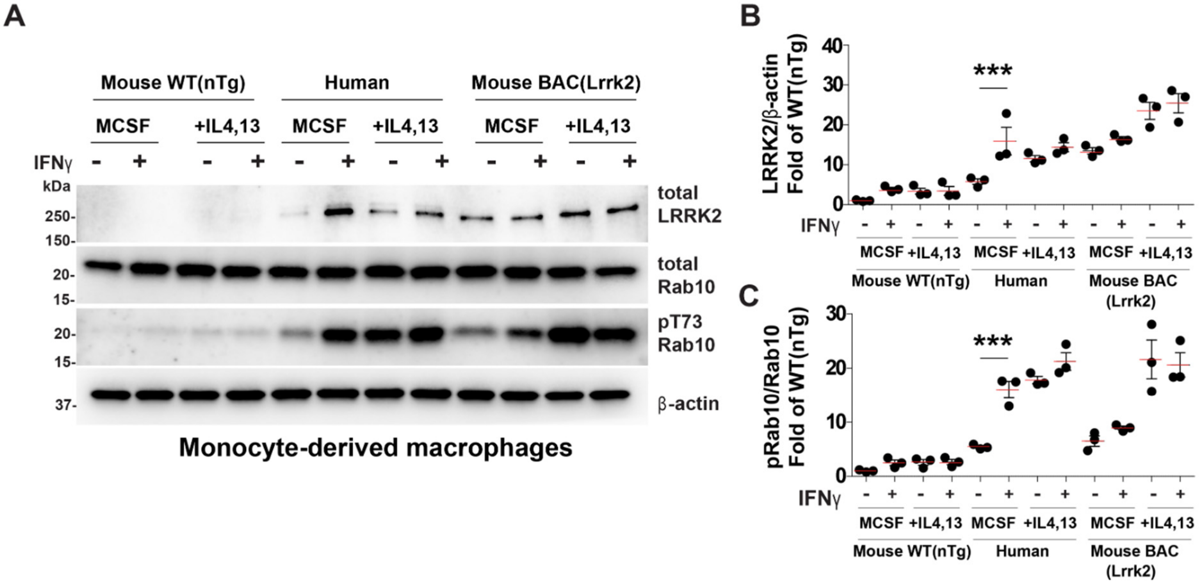

Supplemental Figure 5. LRRK2 expression in human and mouse macrophages (A) Representative immunoblots of lysates from bone marrow-derived macrophages from WT-Lrrk2 non-transgenic (nTg) control mice, human monocyte-derived macrophages, and bone marrow-derived macrophages from WT-Lrrk2 BAC mice. All cells were polarized with MCSF (human or mouse, respectively) in culture for five days and then further treated with or without a combination of IL-4 and IL-13 (human or mouse, respectively) for an additional 48 hours, and treated for 48 hours with IFNγ as indicated (20 ng ・ mL^{-1}). **(B)** Quantification of LRRK2 normalized to β-actin, and **(C)** pT73-Rab10 normalized to total Rab10, calculated from three independent cultures from three male mice each and three healthy male human volunteers. Data are expressed as fold change relative to the WT-Lrrk2 non-transgenic control mice without IFNγ stimulation (first lane of blots in panel A.). Red lines in graphs show group means with error bars depicting ± SEM. Significance is assessed by one-way ANOVA with Tukey's post hoc test, and $***p < 0.001$.

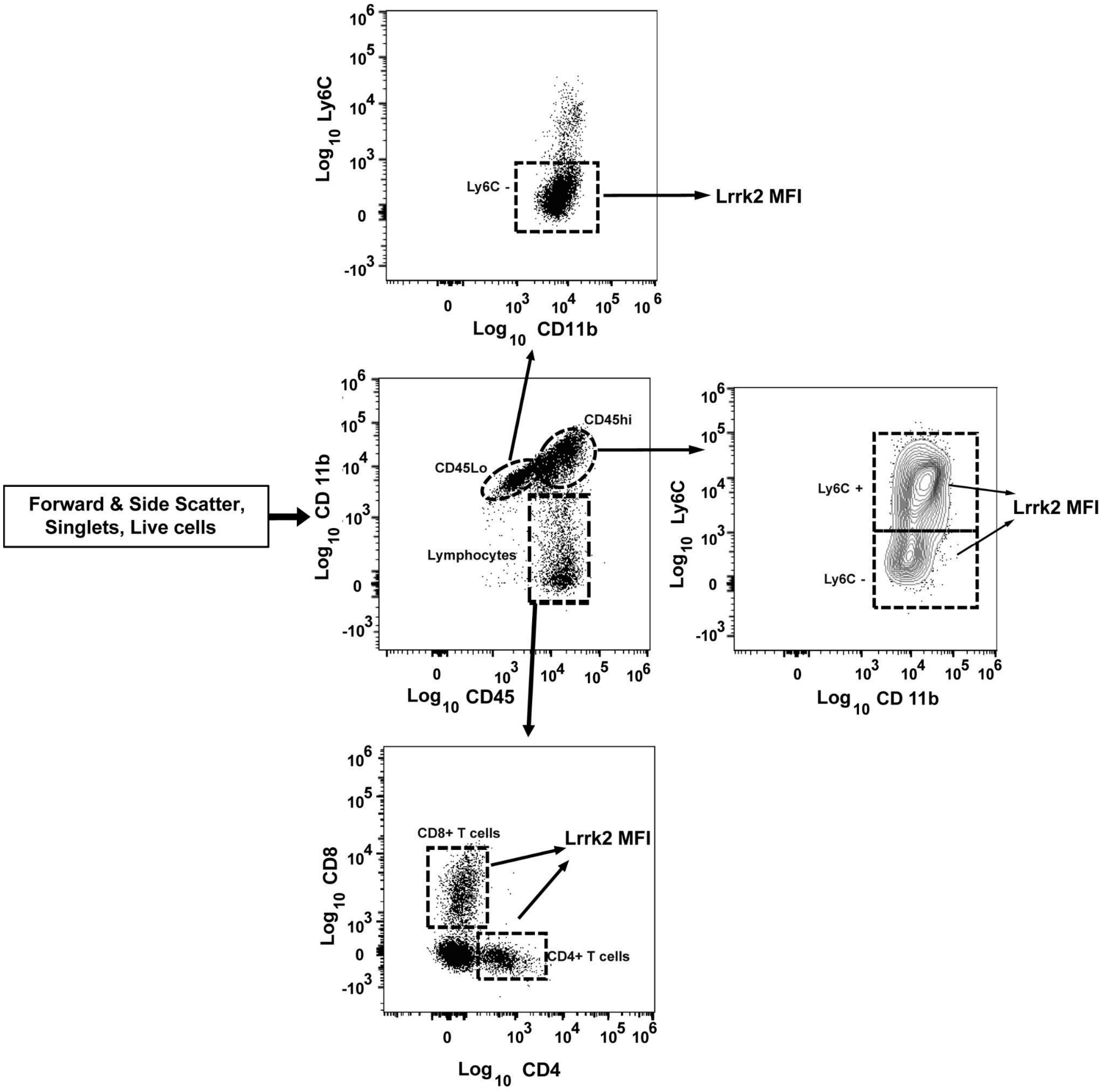

Supplemental Figure 6. Lrrk2 induction in immune cells in the mouse brain after intracranial α-synuclein fibril injection. Flow cytometry gating strategy showing representative cytographs (median fluorescence intensity: MFI, Log_{10}) for live CD45+/CD11b+ cells (after exclusion of debris and doublets) and subsequent gating strategy from a WT-Lrrk2 BAC male mouse midbrain cell homogenates, three days after bilateral injection of 10 µg of α-synuclein h-Fibrils. Injections of matched amounts of α-synuclein fibrils into Lrrk2 knockout (KO) cells were used as controls to assess specific Lrrk2 expression in different immune cell populations. Dashed boxes represent gates, and a representative contour plot is shown for Ly6C+ cells and Ly6C- (neg)

cells after fibril injections. Lrrk2 MFI was analyzed for each gated population *viz*. $CD45^{hi}$, Ly6C+/ Ly6C-, and CD4 T-cells, CD8 T-cells and $CD45^{Lo}$ cells are gated for Ly6C.

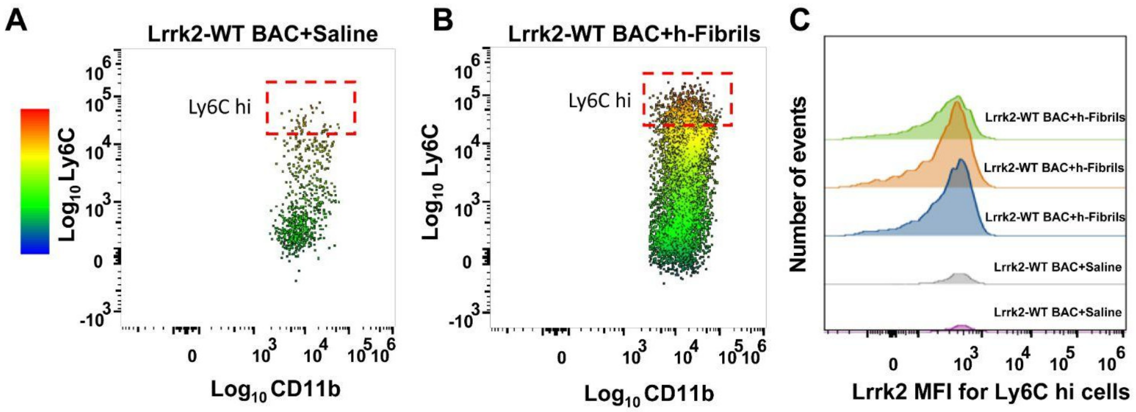

Supplemental Figure 7. Control (vehicle) injections into the mouse midbrain do not result in a significant accumulation of LRRK2-positive CD45^{hi} cells. Representative analysis of CD45^{hi} cells infiltrated into the midbrain as analyzed by flow cytometry 72 hrs after the injection of control (saline) or fibrils into the mouse (Lrrk2-WT BAC) midbrain. **(A)** Scatter plots of CD45^{hi} cells analyzed for CD11b and Ly6C expression for WT-Lrrk2 BAC mice injected with saline or **(B)** human α-synuclein fibrils (h-fibrils). **(C)** Representative histograms compare median-fluorescence intensity (MFI) of cells positive for Lrrk2 (intracellular flow cytometry) from the Ly6C^{hi} monocyte population (gated in red boxes) for the indicated mouse (two mice saline injected, three with fibrils). Consistent with Figure 4, mice were bi-laterally injected in the SNpc. All mice shown here were 2-3 months old male WT-Lrrk2 BAC mice.

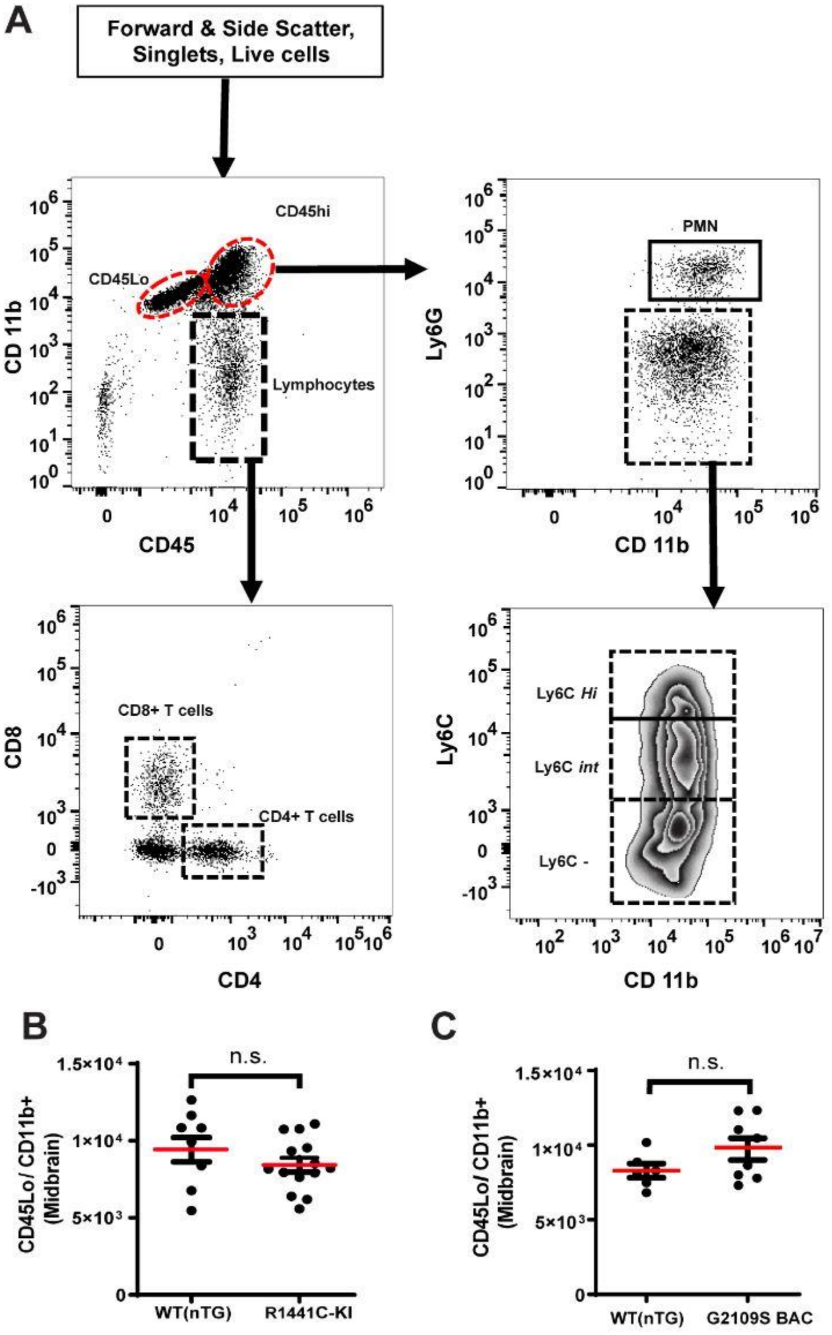

Supplemental Figure 8. Flow cytometry gating strategy for the recruitment of immune cells into the brain and quantification of microglia after α-synuclein fibril injection (A) Representative cytographs (median fluorescence intensity; MFI, Log_{10}) are shown for live CD45+/CD11b+ cells (after exclusion of debris and doublets) and subsequent gating strategy from midbrain cell homogenates three days after injection (bilaterally) with 10 µg of α-synuclein fibrils. Monomer injections of matched amounts of α-synuclein, that fail to recruit monocytes or T-cells,

were used as controls. Dashed/solid line boxes represent gates, and density distributions (zebra plot) are shown for Ly6C+ cells ($Ly6C^{hi}$ and $Ly6C^{int}$ cells) and Ly6C- (neg) cells. $Ly6C^{hi}$ cells in the gates are quantified and graphed in Figure 5. PMN are polymorphonuclear neutrophils. **(B)** & **(C)** Dot plot graphs for quantification of the number of $CD45^{Lo}$/CD11b+ (e.g., microglia) in R1441C-KI and G2019S BAC mice compared to their respective WT (nTG) control mice after 72 hrs of bilateral midbrain α-synuclein fibril injections. Graphs show group means ± SEM. Each dot represents an individual mouse; 22 mice (9 males and 13 females) for Figure B and 14 mice (5 males and 9 females) for Figure C. All mice were aged 2-3 months. Significance is determined by unpaired *t*-test. n.s., not significant.

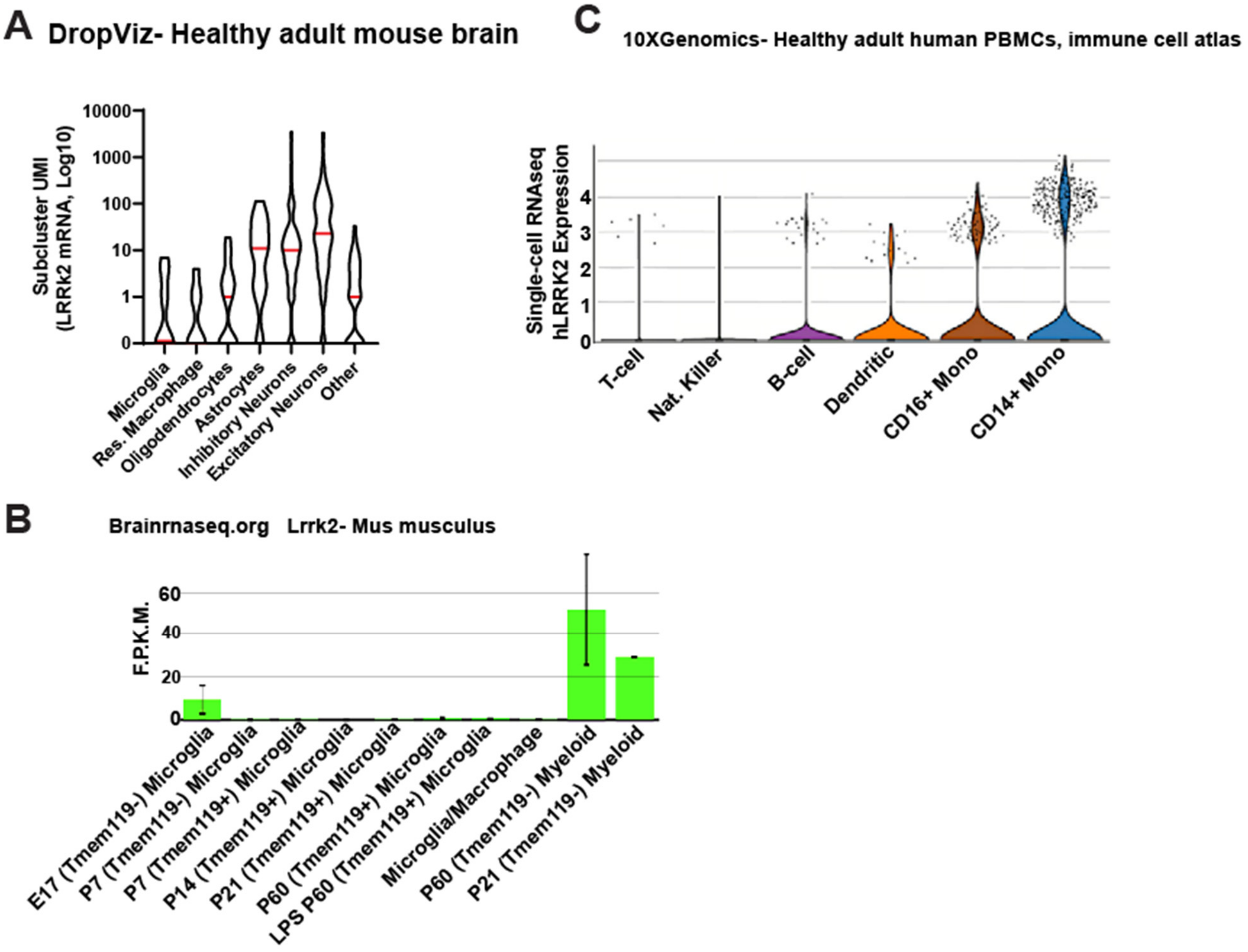

Supplemental Figure 9. Differential LRRK2 expression in healthy mouse brain and immune cells in the blood from healthy volunteers. (A) DropViz (see reference [2]) results for normalized mouse Lrrk2 mRNA expression curated from 690,000 cells sequenced across healthy C57BL/6 adult mouse brains. Labels represent meta-cell averages of subclusters of similar cell types through the brain that include microglia, resident macrophages (e.g., perivascular, meningeal, choroid plexus macrophages), astrocytes, oligodendrocytes, endothelial cells, inhibitory neurons, and excitatory neurons. The meta-cell average of 'other' includes the minority of cells that could not be assigned to these cell types. **(B)** Developmental mouse microglia RNA-Seq. Time points and conditions (LPS exposure) are indicated, data from Zhang et al. [3]. **(C)** Violin plots of normalized single-cell expression of human LRRK2 mRNA from sorted human peripheral blood mononuclear cell isolations from two healthy donors (data.humancellatlas.org/). Meta-categories include T-cells, natural killer cells, B-cells, dendritic cells, and monocytes (further separated into CD16^{hi} and CD14^{hi} populations).

References to Supplemental Figures

1. Ii M, Matsunaga N, Hazeki K, Nakamura K, Takashima K, Seya T, et al. A novel cyclohexene derivative, ethyl (6R)-6-[N-(2-Chloro-4-fluorophenyl)sulfamoyl]cyclohex-1-ene-1-carboxylate (TAK-242), selectively inhibits toll-like receptor 4-mediated cytokine production through suppression of intracellular signaling. Mol Pharmacol. 2006;69:1288–95.
2. Saunders A, Macosko EZ, Wysoker A, Goldman M, Krienen FM, de Rivera H, et al. Molecular diversity and specializations among the cells of the adult mouse brain. Cell. 2018;174:1015–30.e16.
3. Zhang Y, Chen K, Sloan SA, Bennett ML, Scholze AR, O'Keeffe S, et al. An RNA-sequencing transcriptome and splicing database of glia, neurons, and vascular cells of the cerebral cortex. J Neurosci. 2014;34:11929–47.
