# Supplementary figures and images for: Pathological α-synuclein recruits LRRK2 expressing pro-inflammatory monocytes to the brain
Source: Mol Neurodegener. 2022 Jan 10;17:7. doi: 10.1186/s13024-021-00509-5 (PMC8751347; doi:10.1186/s13024-021-00509-5)

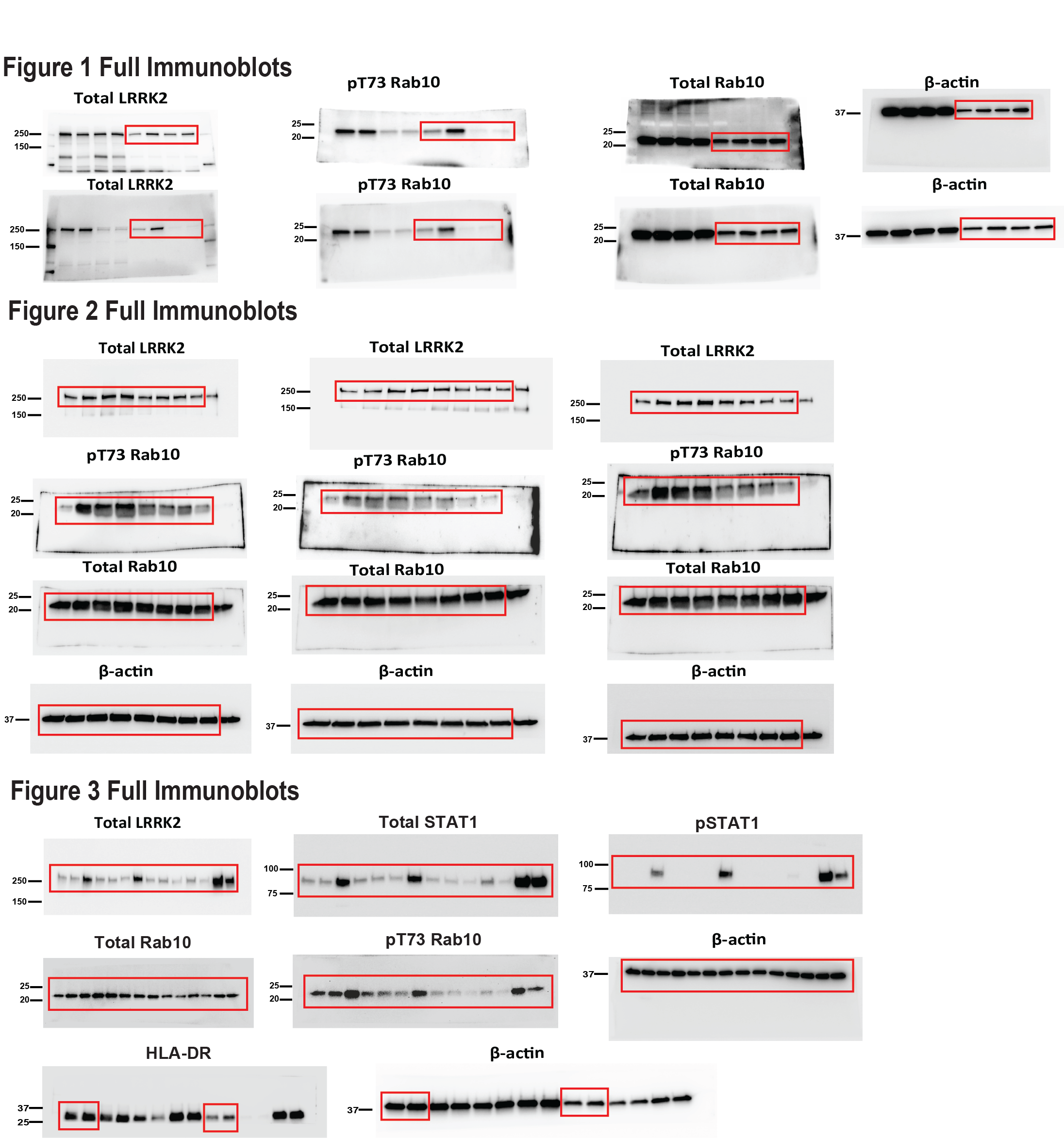

Supplement: Supplementary file 3 — Additional file 3. [file 13024_2021_509_MOESM3_ESM.zip › Full WBs Figs 1-3.png]

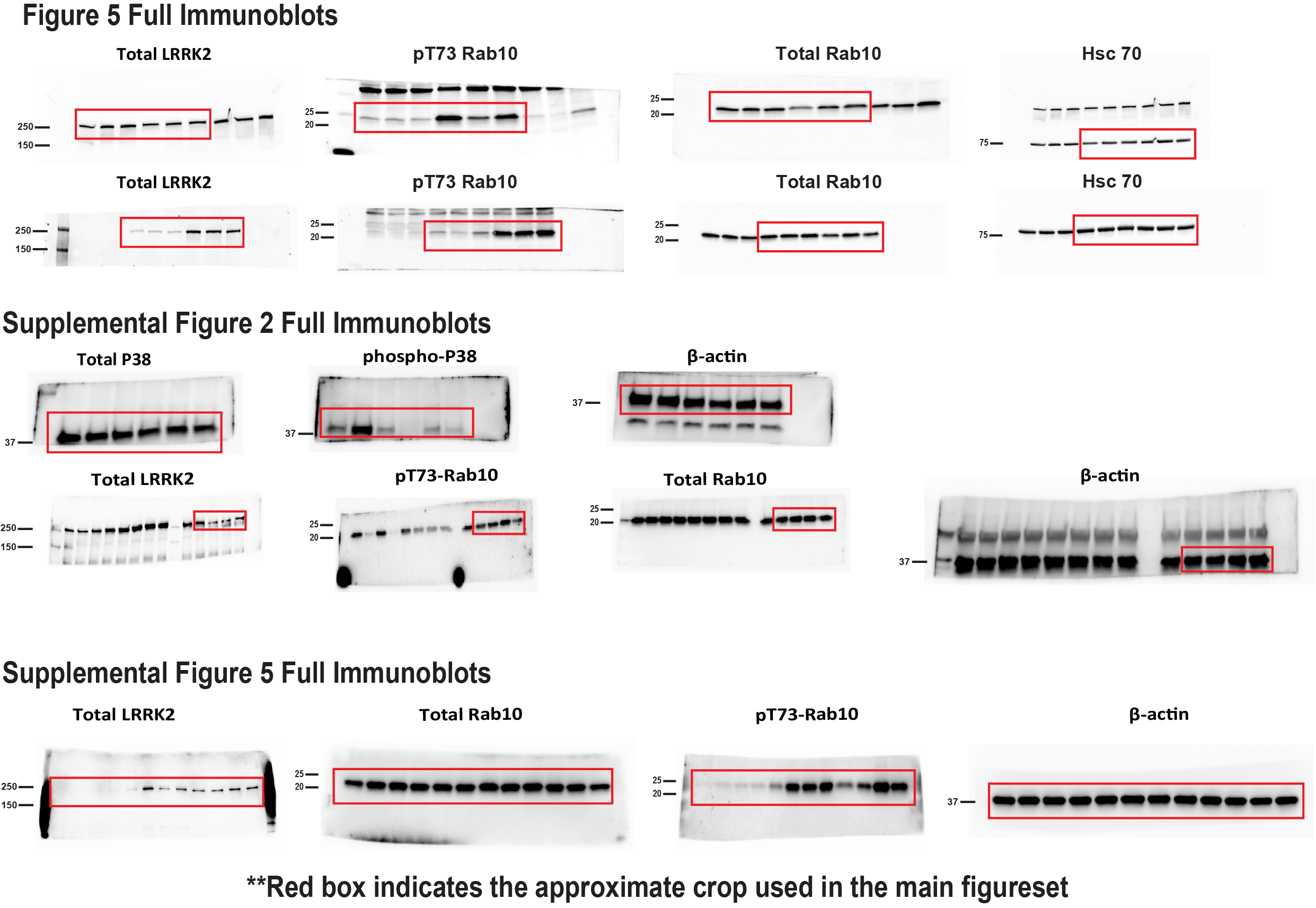

Supplement: Supplementary file 3 — Additional file 3. [file 13024_2021_509_MOESM3_ESM.zip › Full WBs Figs 5 & Supplemental Figs.png]
